# Post-marketing safety profile of cladribine in multiple sclerosis: a disproportionality analysis based on the FDA adverse event reporting system

**DOI:** 10.1007/s11096-025-02041-8

**Published:** 2025-11-15

**Authors:** Yuwen Hu, Jianghai He, Zheng Tu, Hongyu Ye, Caixiang Zhuang, Ziyang Jin, Haoxiang Hu, Yunhan Zhao, Yanyan Zheng, Qiong Yao

**Affiliations:** 1 Department of Neurology, Postgraduate Training Base Alliance of Wenzhou Medical University, Wenzhou People’s Hospital, Wenzhou, 325000 Zhejiang China; 2 Wenzhou Third Clinical Institute Affiliated to Wenzhou Medical University, Wenzhou People’s Hospital, Wenzhou, 325000 Zhejiang China

**Keywords:** Adverse events, Cladribine, Disproportionality analysis, FAERS database, Pharmacovigilance

## Abstract

**Introduction:**

Cladribine has been widely recognized as a therapeutic option for relapsing–remitting multiple sclerosis (MS), but there is still a dearth of real-world data regarding its safety profile.

**Aim:**

This study aimed to assess adverse events (AEs) linked to cladribine in MS patients, utilizing data from the U.S. Food and Drug Administration Adverse Event Reporting System (FAERS).

**Method:**

AE reports identifying cladribine as the primary suspect drug were extracted from the U.S. FAERS, covering the period from the first quarter of 2019 to the third quarter of 2024. Four disproportionality methods—reporting odds ratio (ROR), proportional reporting ratio, Bayesian confidence propagation neural network, and empirical Bayes geometric mean—were employed to evaluate the association between cladribine and AEs. Furthermore, the Weibull distribution model was applied to analyze time-to-onset patterns, and subgroup analyses were conducted based on sex and age.

**Results:**

After screening 4,833 cladribine-related reports, 113 preferred terms (PTs) were identified as positive across all four disproportionality methods. Known AEs such as pneumonia (n = 190, ROR 2.85), lymphopenia (n = 111, ROR 4.19), and drug-induced liver injury (n = 22, ROR 5.94). Unexpected events, including rheumatoid arthritis (ROR 5.64), hypothyroidism (ROR 5.04), eye hemorrhage (ROR 6.88), uveitis (ROR 4.86), retinal detachmen (ROR 4.37), brain edema (ROR 6.12), acute myocardial infarction (ROR 3.70), and completed suicide (ROR 7.46), were also reported. Stratified analysis revealed that females were at a higher risk of nausea, alopecia, and migraine, while males were more susceptible to gait disturbance and sepsis. Older adults (≥ 65 years) faced increased risks of leukopenia and urinary tract infections (UTIs). The median onset of AEs was 152 days, with the highest proportion (28.18%) reported in the first month. Weibull analysis indicated an early peak (shape parameter 0.72).

**Conclusion:**

This study not only corroborates previously established risks associated with cladribine but also uncovers new potential safety signals, highlighting the importance of vigilance for early acute toxicity.

**Supplementary Information:**

The online version contains supplementary material available at 10.1007/s11096-025-02041-8.

## Impact statements

- A FAERS analysis uncovered 113 PT signals for cladribine, confirming known risks like lymphopenia, pneumonia, and drug-induced liver injury, while also revealing unexpected ones such as hypothyroidism and ocular hemorrhage.
- Time-to-onset analysis indicated an early risk peak within the first month of therapy, highlighting the necessity for early safety monitoring.
- Subgroup analyses further revealed distinct risk patterns by sex and age, underscoring the importance of individualized risk assessment, patient counseling, and tailored surveillance strategies in clinical practice.

## Introduction

Multiple sclerosis (MS), a chronic inflammatory disease, severely affects the quality of life and social productivity of young and middle-aged adults. The incidence and prevalence of MS vary significantly by region, with higher latitude populations at greater risk [1]. MS is primarily classified into three clinical subtypes: relapsing–remitting MS (RRMS), primary progressive MS (PPMS), and secondary progressive MS (SPMS), with RRMS accounting for approximately 80–90% of cases at initial diagnosis [2]. Cladribine, approved by the European Medicines Agency (EMA) in 2017 and the U.S. Food and Drug Administration (FDA) in 2019 for relapsing forms of MS, is a novel immune reconstitution therapy (IRT), which selectively reduces autoreactive B cells (especially memory B cells) and T cells, thereby modulating immune responses and delaying disease progression [3]. Clinical data indicate its therapeutic effects can last over four years, offering a unique “treatment-free remission” [4, 5]. Seven-year follow-up data from the MAPLE-MS project also indicate that the incidence of congenital malformations in individuals exposed to cladribine before or during pregnancy is 1.1%, aligning with baseline rates in the general population and supporting its reproductive safety for women of childbearing potential [6].

Though cladribine has proven highly effective in treating MS, its immunomodulatory properties also raise potential systemic safety issues. Current clinical data indicate that lymphocytopenia is the most notable adverse event (AE) associated with this agent [7]. Recent pharmacovigilance studies have pointed to an increased risk of herpes zoster with cladribine use [8]. Additionally, studies utilizing the European spontaneous reporting system have confirmed that cladribine poses significant safety risks to the hepatobiliary system, underscoring the need for close monitoring in patients receiving treatment [9]. However, most existing pharmacovigilance evidence comes from isolated studies focusing on specific safety risks [8, 9].

A recent analysis of the Italian drug monitoring database provided a broad overview of adverse reactions across MS therapies, yet its coverage of various drugs led to limited focus on cladribine [10]. Additionally, its key analysis was confined to a small subset of reports from Sicily, resulting in a very small sample size. Therefore, a systematic evaluation of cladribine’s overall safety profile, utilizing a large-scale database and robust methodologies, is still absent.

To bridge this gap, this study turned to the FDA Adverse Event Reporting System (FAERS) database, a publicly accessible spontaneous reporting database that serves as a vital source of real-world evidence for post-marketing safety monitoring and risk assessment [11]. We applied multiple disproportionality analysis methods to conduct a comprehensive pharmacovigilance evaluation of AEs associated with cladribine in MS treatment. The findings are intended to inform evidence-based risk minimization strategies and promote rational clinical use of the drug.

### Aim

This study aimed to assess AEs related to cladribine in the treatment of MS using data from the U.S. FAERS.

## Method

### Data sources and data cleaning

This study adhered to the Reporting of a Disproportionality Analysis for Drug Safety Signal Detection Using Individual Case Safety Reports in Pharmacovigilance (READUS-PV) guidelines [12]. We performed a retrospective pharmacovigilance analysis of cladribine, an MS therapy, using FAERS data (https://fis.fda.gov/extensions/FPD-QDE-FAERS/FPD-QDE-FAERS.html). The study spanned all AE reports from the drug’s FDA approval in the first quarter of 2019 through the third quarter of 2024. Data were extracted from crucial FAERS subfiles, which included demographic details (DEMO), drug information (DRUG), adverse event reports (REAC), patient outcomes (OUTC), reporting sources (RPSR), therapy dates (THER), and indications for use (INDI).

**Fig. 1:**
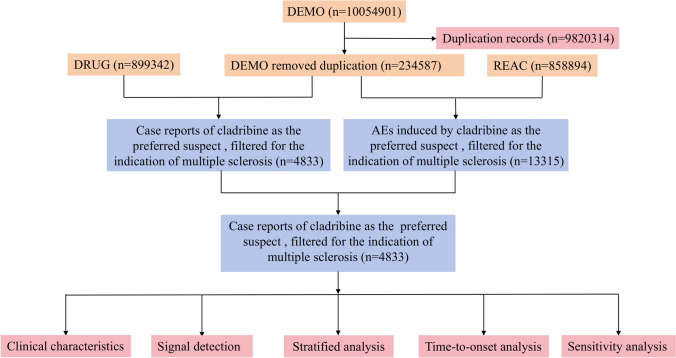
Flow diagram (DEMO demographic and administrative information, DRUG drug information, REAC preferred terminology for adverse event

Case identification involved querying the DRUG file for records listing cladribine (generic name) or Mavenclad (brand name) as the ‘Primary Suspect’ (PS), restricted to the indication of multiple sclerosis. Data cleaning followed FDA-recommended procedures. Specifically, duplicate reports were removed by retaining the most recent submission for each unique case identification number (CASEID) based on the FDA received date (FDA_DT), with the higher primary identification number (PRIMARYID) used to resolve ties. To concentrate on adverse drug reactions, we excluded PTs related to medication and product issues (e.g., “product error”, “medication error”, “overdose”) and reports with the indication “product used for unknown indication”. All AEs were coded using the Medical Dictionary for Regulatory Activities (MedDRA 27.1), which classifies events into PTs and System Organ Classes (SOCs). Each individual case safety report (ICSR) could encompass multiple PTs, and all reported PTs were included in the subsequent disproportionality analysis. We extracted available clinical characteristics from the reports, including patient demographics (gender, age), report origin, outcomes, and reporter type. The overall data processing workflow is detailed in Fig. 1.

### Statistical analysis

Disproportionality analysis methods, including reporting odds ratio (ROR), proportional reporting ratio (PRR), Bayesian confidence propagation neural network (BCPNN), and empirical Bayes geometric mean (EBGM), were employed for signal detection to comprehensively evaluate AEs linked to cladribine treatment for MS. The calculation formulas are provided in Supplementary Table 1. Compared to ROR, PRR offers higher sensitivity but is more susceptible to false positives [13]. BCPNN helps mitigate biases from relying on a single data source. EBGM is useful for assessing the strength of the association between drugs and AEs, especially in identifying rare signals [14, 15]. For each PT, we constructed a 2 × 2 table (detailed in Supplementary Table 2) comparing cladribine reports against all non-cladribine FAERS reports and computed ROR, PRR, IC, and EBGM. A positive signal was strictly defined as an AE flagged concurrently by all four methodologies and reported in at least three cases, effectively reducing false positives. Furthermore, this study used the chi-square test to compare differences in adverse reactions across gender and age groups and explored the impact of these factors on drug safety through stratified analysis. Additionally, time trend analysis methods, including onset time assessment and Weibull distribution analysis, were employed to examine the temporal characteristics of adverse reactions, offering valuable insights into potential long-term safety risks. Data mining and statistical analyses, including chi-square tests, were primarily conducted using R software (version 4.4.1).

### Within-indication comparative analysis

A direct comparison analysis was conducted on the safety signals of cladribine and fingolimod (another oral disease-modifying therapy for MS). To ensure comparability, adverse event reports for both drugs were extracted from the same time period (Q1 2019 to Q3 2024) and analyzed using the same four disproportionality methods (ROR, PRR, BCPNN, and EBGM). Each drug’s relevant reports were strictly screened from the adverse drug reaction reporting system based on two criteria: (1) The drug was classified as the “primary suspect” and indicated for “MS”. (2) Reports indicating concurrent use of another study drug were excluded. The imbalances generated by each purified group were then compared to highlight differences in AE characteristics.

### Sensitivity analysis

To assess the potential impact of concomitant medications and reporting sources on the study results, two independent sensitivity analyses were performed using stringent data selection criteria: (1) Exclusion of cases involving frequently reported, high-risk concomitant medications. (2) Reports strictly limited to those submitted by healthcare professionals.

### Ethics approval

This research did not involve individual-level data, thus eliminating the need for new ethical approval from an ethics review board.

## Results

### General characteristics

Based on the FAERS database, from the first quarter of 2019, when cladribine gained US FDA approval for MS treatment, through the third quarter of 2024, a total of 4,833 reports identified cladribine as the “primary suspected” drug (Table 1). Female patients accounted for a notably larger share of these reports (75.8%) compared to males (21.4%). After excluding 1,990 reports with missing age data, the majority of patients (53.7%) fell within the 18–64.9 age range, followed by those aged 65–85 years (4.8%). The year 2023 recorded the highest number of AE reports, totaling 1,132. These reports came from 64 countries, with the United States (59.3%), Canada (5.4%), the United Kingdom (5.4%), Germany (5.0%), and Australia (3.3%) being the top five contributors. Consumers (63.0%) and physicians (23.5%) submitted most of the reports. Serious outcomes were noted in 3,045 cases (63.0%), including 132 deaths (2.7%), 35 disabilities (0.7%), 69 life-threatening events (1.4%), 919 hospitalizations (19.0%), 8 congenital anomalies (0.2%), and 1,882 other serious outcomes (38.9%).

**Table 1:** Clinical characteristics of adverse events associated with cladribine treatment for multiple sclerosis based on the FAERS database

Number of reports	Cladribine
	(N = 4833)
Sex	
Female	3664 (75.8%)
Male	1036 (21.4%)
Missing	133 (2.8%)
Weight (kg)	
< 50	39 (0.8%)
> 100	75 (1.6%)
50–100	470 (9.7%)
Missing	4249 (87.9%)
Age (year)	
< 18	16 (0.3%)
18–64.9	2594 (53.7%)
65–85	233 (4.8%)
Missing	1990 (41.2%)
Reporting Year	
2019 year	463 (9.6%)
2020 year	649 (13.4%)
2021 year	837 (17.3%)
2022 year	923 (19.1%)
2023 year	1132 (23.4%)
2024 year	829 (17.2%)
Reporter type	
Consumer	3043 (63.0%)
Health Professional	479 (9.9%)
Physician	1138 (23.5%)
Other Health Professional	38 (0.8%)
Pharmacist	125 (2.6%)
Missing	10 (0.2%)
Reporter Country	
United States	2865 (59.3%)
United Kingdom	260 (5.4%)
Canada	260 (5.4%)
Germany	242 (5.0%)
Australia	159 (3.3%)
Other Country	1047 (21.7%)
Outcome	
Death	132 (2.7%)
Disability	35 (0.7%)
Life-Threatening	69 (1.4%)
Hospitalization	919 (19.0%)
Congenital Anomaly	8 (0.2%)
Recurrent Issue	1 (0.0%)
Other Serious Outcome	1882 (38.9%)
Missing	1787 (37.0%)

### Disproportionality analysis

Supplementary Table 3 outlines the distribution of cladribine-related AE reports at the SOC level. Signal detection analysis revealed 27 SOCs associated with cladribine. The highest number of AEs was reported for nervous system disorders (n = 2089), followed by infections and infestations (n = 1719), general disorders and administration site conditions (n = 1706), investigations (n = 1518), and gastrointestinal disorders (n = 969). Notably, despite having relatively low report frequencies, blood and lymphatic system disorders (n = 282), pregnancy, puerperium, and perinatal conditions (n = 181), and endocrine disorders (n = 91) displayed notably high signal strengths and were the only categories meeting positive signal criteria across all four algorithms.

Following statistical analysis and exclusion of non-treatment-related signals, a total of 113 AEs signals meeting criteria across all four algorithms were identified for cladribine (see Supplementary Table 4 for details). As listed in Table 2, which ranks the top 50 AEs by frequency, the most commonly reported AEs included decreased lymphocyte count (ROR 6.34), decreased white blood cell count (ROR 3.82), and pneumonia (ROR 2.85), among others. When ranked by ROR signal strength, the top five AEs were pertussis (ROR 22.94), polyarthritis (ROR 16.25), complication of pregnancy (ROR 16.25), dysmenorrhea (ROR 13.82), and viral pneumonia (ROR 12.64).

**Table 2:** Top 50 adverse events of cladribine in the FAERS database

PT	Case reports	ROR (95%Cl)	PRR(χ^2^)	EBGM(EBGM05)	IC(IC025)
Lymphocyte count decreased	336	6.34 (5.66–7.10)	6.21 (1345.73)	5.75 (5.23)	2.52 (2.36)
White blood cell count decreased	236	3.82 (3.35–4.36)	3.77 (456.64)	3.62 (3.24)	1.86 (1.66)
Pneumonia	190	2.85 (2.46–3.30)	2.83 (215.87)	2.75 (2.43)	1.46 (1.24)
Lymphopenia	111	4.19 (3.45–5.08)	4.16 (250.99)	3.97 (3.38)	1.99 (1.71)
Lower respiratory tract infection	71	6.58 (5.15–8.41)	6.55 (303.64)	6.04 (4.92)	2.60 (2.24)
Alanine aminotransferase increased	60	3.61 (2.78–4.69)	3.60 (106.83)	3.46 (2.78)	1.79 (1.41)
Aspartate aminotransferase increased	48	4.60 (3.43–6.16)	4.59 (125.79)	4.35 (3.4)	2.12 (1.69)
Nephrolithiasis^*^	48	2.64 (1.98–3.52)	2.63 (46.72)	2.57 (2.02)	1.36 (0.94)
Kidney infection	39	4.08 (2.95–5.64)	4.07 (85.15)	3.89 (2.97)	1.96 (1.49)
Platelet count decreased	37	3.84 (2.76–5.35)	3.83 (73.22)	3.68 (2.78)	1.88 (1.40)
Hypothyroidism^*^	32	5.04 (3.52–7.23)	5.03 (96.03)	4.74 (3.51)	2.25 (1.72)
Leukopenia	32	2.89 (2.03–4.12)	2.88 (37.72)	2.8 (2.08)	1.49 (0.97)
Neutrophil count decreased	31	6.13 (4.24–8.87)	6.12 (121.42)	5.68 (4.17)	2.51 (1.97)
Diverticulitis	30	5.55 (3.82–8.06)	5.54 (102.79)	5.18 (3.79)	2.37 (1.83)
Drug-induced liver injury	22	5.94 (3.84–9.19)	5.93 (82.65)	5.52 (3.83)	2.46 (1.84)
Haematochezia^*^	22	5.09 (3.30–7.86)	5.09 (66.99)	4.79 (3.33)	2.26 (1.64)
Rheumatoid arthritis^*^	21	5.64 (3.61–8.82)	5.64 (73.72)	5.27 (3.63)	2.40 (1.76)
Hypophagia^*^	19	5.80 (3.63–9.28)	5.79 (69.22)	5.40 (3.65)	2.43 (1.76)
Thrombocytopenia	19	3.64 (2.30–5.79)	3.64 (34.47)	3.50 (2.38)	1.81 (1.14)
Dysmenorrhoea^*^	17	13.82 (8.19–23.33)	13.80 (166.53)	11.56 (7.46)	3.53 (2.79)
Blood potassium decreased^*^	17	3.15 (1.93–5.12)	3.15 (23.75)	3.05 (2.03)	1.61 (0.91)
Cyst^*^	15	4.20 (2.49–7.09)	4.20 (34.36)	4.01 (2.59)	2.00 (1.26)
Blood bilirubin increased	14	4.03 (2.35–6.91)	4.02 (29.97)	3.85 (2.45)	1.94 (1.18)
Autoimmune thyroiditis^*^	12	4.29 (2.39–7.69)	4.28 (28.34)	4.08 (2.50)	2.03 (1.20)
Eye haemorrhage^*^	11	6.88 (3.69–12.8)	6.87 (49.91)	6.31 (3.75)	2.66 (1.78)
Unresponsive to stimuli^*^	11	4.61 (2.50–8.51)	4.61 (29.04)	4.37 (2.62)	2.13 (1.27)
Premature baby	10	7.47 (3.88–14.38)	7.47 (50.24)	6.80 (3.93)	2.77 (1.85)
Angioedema	9	4.24 (2.16–8.32)	4.24 (20.89)	4.04 (2.3)	2.01 (1.07)
Acute myocardial infarction^*^	9	3.70 (1.89–7.25)	3.70 (16.78)	3.55 (2.03)	1.83 (0.89)
Complication of pregnancy	8	16.25 (7.49–35.27)	16.24 (91.53)	13.19 (6.90)	3.72 (2.66)
Graves' disease^*^	8	10.40 (4.93–21.94)	10.39 (58.55)	9.10 (4.87)	3.19 (2.15)
Myelodysplastic syndrome	8	8.81 (4.21–18.45)	8.81 (48.76)	7.88 (4.24)	2.98 (1.95)
Brain oedema^*^	8	6.12 (2.96–12.63)	6.11 (31.28)	5.67 (3.09)	2.50 (1.50)
Cervical dysplasia^*^	8	5.00 (2.43–10.26)	5.00 (23.75)	4.71 (2.58)	2.24 (1.24)
Uveitis^*^	8	4.86 (2.37–9.97)	4.86 (22.8)	4.59 (2.51)	2.20 (1.20)
Faeces discoloured^*^	8	4.77 (2.33–9.78)	4.77 (22.19)	4.51 (2.47)	2.17 (1.18)
Pneumonia viral	7	12.64 (5.62–28.40)	12.63 (62.77)	10.74 (5.45)	3.42 (2.32)
Completed suicide^*^	7	7.46 (3.41–16.31)	7.45 (35.09)	6.79 (3.53)	2.76 (1.69)
Subcutaneous abscess	7	6.23 (2.87–13.54)	6.23 (28.04)	5.77 (3.02)	2.53 (1.46)
Pneumonia bacterial	7	4.59 (2.13–9.89)	4.59 (18.38)	4.36 (2.29)	2.12 (1.07)
Retinal detachment^*^	7	4.37 (2.03–9.40)	4.37 (17.06)	4.16 (2.19)	2.06 (1.00)
Pertussis	6	22.94 (9.04–58.18)	22.93 (93.00)	17.21 (7.90)	4.10 (2.87)
Polyarthritis^*^	6	16.25 (6.64–39.75)	16.24 (68.65)	13.19 (6.24)	3.72 (2.51)
Metastases to central nervous system^*^	6	9.28 (3.95–21.84)	9.28 (38.79)	8.24 (4.03)	3.04 (1.88)
Blood creatine phosphokinase increased^*^	6	6.96 (3.00–16.16)	6.96 (27.66)	6.38 (3.16)	2.67 (1.52)
Hepatitis c	6	6.39 (2.76–14.79)	6.39 (24.83)	5.91 (2.93)	2.56 (1.42)
Synovial cyst^*^	6	5.34 (2.32–12.28)	5.34 (19.55)	5.01 (2.50)	2.32 (1.19)
Arthritis infective	6	4.94 (2.15–11.32)	4.93 (17.49)	4.66 (2.32)	2.22 (1.08)
Pericarditis^*^	6	4.75 (2.08–10.89)	4.75 (16.57)	4.50 (2.25)	2.17 (1.04)
Plasma cell myeloma	6	4.59 (2.00–10.5)	4.59 (15.71)	4.35 (2.17)	2.12 (0.99)

*PT* Preferred Terms, *ROR* Reporting Odds Ratio, *PRR* Proportional Reporting Ratio, *χ*^2^, Chi-square test value, *EBGM* Empirical Bayesian Geometric Mean, *EBGM05* the lower limit of the 95% confidence interval of EBGM, *IC* Information Component, *IC025* the lower limit of the 95% confidence interval of IC; CI, Confidence Interval

*The PT is not listed on the label of cladribine

Half of the adverse reactions aligned with those described in the cladribine prescribing information. Blood and lymphatic system disorders, such as lymphopenia, leukopenia, and thrombocytopenia, were particularly prominent. Other labeled events included drug-induced liver injury, pregnancy complications, and angioedema. Infection risks highlighted in the drug label were strongly supported by this study, with significant signals detected for pneumonia, kidney infections, diverticulitis, subcutaneous abscess, hepatitis C, and infective arthritis.

Malignancy and teratogenicity risks, emphasized in black box warnings, were also reflected through signals like myelodysplastic syndrome, plasma cell myeloma, premature birth, and pregnancy complications. Additionally, several unexpected AEs were identified, including decreased blood potassium (ROR 3.15), hypothyroidism (ROR 5.04), autoimmune thyroiditis (ROR 4.29), Graves’ disease (ROR 10.40), eye hemorrhage (ROR 6.88), uveitis (ROR 4.86), retinal detachment (ROR 4.37), unresponsiveness to stimuli (ROR 4.61), brain edema (ROR 6.12), synovial cyst (ROR 5.34), dysmenorrhea (ROR 13.82), cervical dysplasia (ROR 5.00), hematochezia (ROR 5.09), rheumatoid arthritis (ROR 5.64), polyarthritis (ROR 16.25), acute myocardial infarction (ROR 3.70), nephrolithiasis (ROR 2.64), and completed suicide (ROR 7.46). Overall, these findings indicate that cladribine is associated with a wide range of adverse reactions, including several not currently listed in its prescribing information.

### Stratified analysis

Stratified analysis revealed that women treated with cladribine were more likely to experience nausea, decreased white blood cell count, alopecia, and migraine compared to men. Conversely, men had a higher risk of gait disturbance and sepsis. Individuals aged 65 and older were more prone to decreased white blood cell count, UTIs, and falls compared to those aged 18–64.9 years. In contrast, individuals in the 18–64.9 age group were more susceptible to nausea (Table 3).

**Table 3:** Comparison of subgroup risks by sex and age for adverse events of cladribine in multiple sclerosis treatment

PT	Gender				Age			
	Male (N = 2741)	Female (N = 10,375)	ROR (95%)	P-value	≥ 65 years (N = 788)	18–64.9 years (N = 8190)	ROR (95%)	P-value
Fatigue	92	425	0.81 (0.65–1.02)	0.08^a^	24	354	0.7 (0.46–1.06)	0.09^a^
Headache	77	343	0.85 (0.66–1.09)	0.19^a^	16	289	0.57 (0.34–0.94)	< 0.05^a^
Lymphocyte count decreased	58	267	0.82 (0.61–1.09)	0.17^a^	16	205	0.81 (0.48–1.35)	0.41^a^
Nausea	30	221	0.51 (0.35–0.75)	< 0.001^a^	7	185	0.39 (0.18–0.83)	< 0.05^a^
White blood cell count decreased	33	201	0.62 (0.43–0.89)	< 0.05^a^	26	119	2.31 (1.5–3.56)	< 0.001^a^
Pneumonia	44	146	1.14 (0.81–1.61)	0.44^a^	15	103	1.52 (0.88–2.63)	0.13^a^
Urinary tract infection	37	144	0.97 (0.68–1.4)	0.88^a^	16	88	1.91 (1.11–3.27)	< 0.05^a^
Asthenia	29	97	1.13 (0.75–1.72)	0.56^a^	10	95	1.1 (0.57–2.11)	0.79^a^
Dizziness	20	92	0.82 (0.51–1.33)	0.43^a^	8	77	1.08 (0.52–2.25)	0.84^a^
Pain	18	89	0.76 (0.46–1.27)	0.3^a^	8	75	1.11 (0.53–2.31)	0.78^a^
Lymphopenia	24	80	1.14 (0.72–1.8)	0.58^a^	3	65	0.48 (0.15–1.52)	0.2^a^
Diarrhoea	15	77	0.74 (0.42–1.28)	0.28^a^	8	58	1.44 (0.68–3.02)	0.34^a^
Fall	27	74	1.38 (0.89–2.16)	0.15^a^	15	60	2.63 (1.49–4.65)	< 0.001^a^
Vomiting	13	66	0.74 (0.41–1.35)	0.33^a^	4	57	0.73 (0.26–2.01)	0.54^a^
Hypoaesthesia	19	63	1.14 (0.68–1.91)	0.61^a^	5	57	0.91 (0.36–2.28)	0.84^a^
Arthralgia	11	60	0.69 (0.36–1.32)	0.26^a^	7	37	1.97 (0.88–4.44)	0.1^b^
Malaise	13	60	0.82 (0.45–1.49)	0.51^a^	5	34	1.53 (0.6–3.93)	0.39^b^
Feeling abnormal	7	58	0.46 (0.21–1)	< 0.05^a^	7	41	1.78 (0.8–3.98)	0.19^b^
Nasopharyngitis	11	58	0.72 (0.38–1.37)	0.31^a^	1	44	0.24 (0.03–1.71)	0.18^b^
Gait disturbance	32	58	2.1 (1.36–3.24)	< 0.001^a^	7	51	1.43 (0.65–3.16)	0.37^a^
Lower respiratory tract infection	15	55	1.03 (0.58–1.83)	0.91^a^	1	36	0.29 (0.04–2.1)	0.25^b^
Dyspnoea	8	55	0.55 (0.26–1.15)	0.11^a^	4	39	1.07 (0.38–2.99)	0.79^b^
Alopecia	4	53	0.28 (0.1–0.79)	< 0.05^a^	NA	NA	/	/
Weight decreased	18	53	1.29 (0.75–2.2)	0.35^a^	3	36	0.87 (0.27–2.82)	1^b^
Muscular weakness	15	51	1.11 (0.63–1.98)	0.71^a^	6	45	1.39 (0.59–3.27)	0.45^b^
Back pain	9	51	0.67 (0.33–1.36)	0.26^a^	3	42	0.74 (0.23–2.4)	0.79^b^
Decreased appetite	8	50	0.6 (0.29–1.28)	0.18^a^	4	44	0.94 (0.34–2.64)	1^b^
Migraine	3	48	0.24 (0.07–0.76)	< 0.05^a^	2	32	0.65 (0.16–2.71)	0.76^b^
Alanine aminotransferase increased	13	47	1.05 (0.57–1.94)	0.88^a^	NA	NA	/	/
Herpes zoster	9	46	0.74 (0.36–1.51)	0.41^a^	NA	NA	/	/
Loss of consciousness	12	45	1.01 (0.53–1.91)	0.98^a^	3	37	0.84 (0.26–2.74)	1^b^
Rash	5	45	0.42 (0.17–1.06)	0.06^a^	5	25	2.09 (0.8–5.46)	0.18^b^
Pyrexia	17	44	1.47 (0.84–2.57)	0.18^a^	2	45	0.46 (0.11–1.9)	0.43^b^
Paraesthesia	12	43	1.06 (0.56–2.01)	0.87^a^	1	41	0.25 (0.03–1.84)	0.18^b^
Abdominal discomfort	9	43	0.79 (0.39–1.63)	0.52^a^	2	35	0.59 (0.14–2.47)	0.77^b^
Pain in extremity	12	41	1.11 (0.58–2.11)	0.75^a^	6	34	1.84 (0.77–4.4)	0.16^b^
Insomnia	13	38	1.3 (0.69–2.44)	0.42^a^	4	31	1.34 (0.47–3.81)	0.54^b^
Condition aggravated	6	38	0.6 (0.25–1.41)	0.24^a^	NA	NA	/	/
Influenza	5	37	0.51 (0.2–1.3)	0.15^a^	2	22	0.94 (0.22–4.03)	1^b^
Aspartate aminotransferase increased	11	36	1.16 (0.59–2.28)	0.67^a^	NA	NA	/	/
Sepsis	25	36	2.64 (1.58–4.41)	< 0.001^a^	NA	NA	/	/
Seizure	10	35	1.08 (0.54–2.19)	0.83^a^	3	29	1.08 (0.33–3.54)	0.76^b^
Pruritus	4	35	0.43 (0.15–1.22)	0.1^a^	2	31	0.67 (0.16–2.8)	1^b^
Weight increased	8	35	0.86 (0.4–1.87)	0.71^a^	3	25	1.25 (0.38–4.14)	0.73^b^
Platelet count decreased	4	33	0.46 (0.16–1.29)	0.13^a^	NA	NA	/	/
Abdominal pain upper	9	33	1.03 (0.49–2.16)	0.93^a^	2	31	0.67 (0.16–2.8)	1^b^
Infection	9	33	1.03 (0.49–2.16)	0.93^a^	2	23	0.9 (0.21–3.84)	1^b^
Cough	5	33	0.57 (0.22–1.47)	0.24^a^	2	24	0.87 (0.2–3.67)	1^b^
Abdominal pain	3	32	0.35 (0.11–1.16)	0.07^a^	NA	NA	/	/
Anxiety	6	31	0.73 (0.31–1.76)	0.48^a^	2	24	0.87 (0.2–3.67)	1^b^
Myocardial infarction	NA	NA	/	/	4	31	1.34 (0.47–3.81)	0.54^b^
Balance disorder	NA	NA	/	/	3	30	1.04 (0.32–3.41)	0.76^b^
Myalgia	NA	NA	/	/	1	27	0.38 (0.05–2.83)	0.51^b^
Chest pain	NA	NA	/	/	3	26	1.2 (0.36–3.97)	0.74^b^
Muscle spasms	NA	NA	/	/	1	25	0.41 (0.06–3.07)	0.72^b^
Nephrolithiasis	NA	NA	/	/	3	24	1.3 (0.39–4.33)	0.73^b^
Influenza like illness	NA	NA	/	/	2	23	0.9 (0.21–3.84)	1^b^
Vision blurred	NA	NA	/	/	2	22	0.94 (0.22–4.03)	1^b^

*PT* Preferred Terms, *N* Number of cases, *ROR* reporting odds ratio, *CI* confidence interval

The listed AEs had significant signal strengths, as assessed by reporting odds ratios (ROR) with 95% confidence intervals

^a^Pearson chi-square test

^b^Fisher’s exact test

P-value < 0.05 were considered statistically significant

### Time-to-onset analysis

**Fig. 2:**
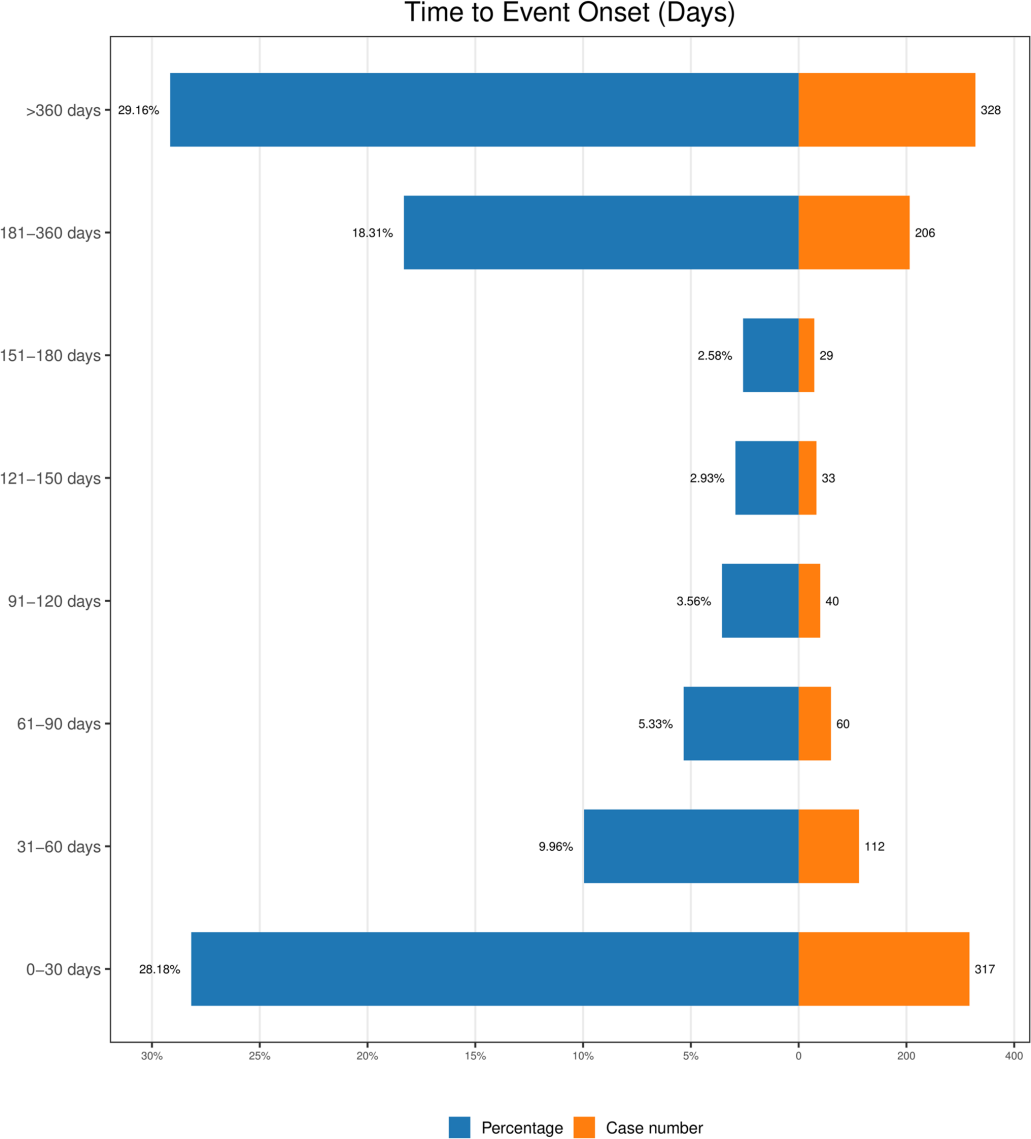
Onset time distribution of adverse events associated with cladribine

We gathered data on the onset time of MS-related AEs in patients treated with cladribine from the database, excluding cases with missing or undisclosed onset times. A total of 1,125 reports provided onset time information, with a median onset time of 152 days (IQR 27–385). As depicted in Fig. 2, the first peak in reporting proportion (28.18%) occurred during the first month of treatment, followed by a gradual decline. Weibull distribution analysis revealed a shape parameter of 0.72 (95% CI 0.68–0.75), indicating that AEs primarily occurred early in treatment (Table 4).

**Table 4:** Time to onset of cladribine-associated adverse events and Weibull distribution analysis

Cases	TTO (days) and Weibull distribution test						
	Median/(IQR)	Min–Max	Scale Parameter	95% CI for α	Shape Parameter	95% CI for β	Failure Type
1125	152 (27–385)	1–3097	208.42	190.59–226.25	0.72	0.68–0.75	Early failure

*TTO* time to onset, *CI* confidence interval, *IQR* interquartile range

### Comparison of safety signals between cladribine and fingolimod

A comparative study was conducted on cladribine and fingolimod within the same group to clarify their safety profiles (see Supplementary Tables 5 and 6). Both agents demonstrated signals for hematological toxicity. In contrast, fingolimod was linked to stronger signals for ocular disorders (most notably macular edema) and skin/subcutaneous tissue disorders (with skin hemangioma being the strongest signal), while cladribine was associated with a broader range of infections.

### Sensitivity analysis

Applying the selection criteria, this study identified 4756 cladribine-treated MS patients without frequent, high-risk concomitant medications and 1768 MS patients reported exclusively by healthcare professionals. Overall, the majority of signals demonstrated good robustness. Among the 50 initially identified signals, all remained significant after excluding high-risk concomitant medications, and 34 of these signals continued to show significance in the subgroup limited to HCP reports. It is worth noting that 16 signals did not reach significance in the HCP subgroup, primarily due to data limitations resulting from an insufficient number of reported cases (n < 3). Furthermore, no new significant signals were identified in either sensitivity analysis. Detailed results are provided in Supplementary Table 7.

## Discussion

This study utilized the FAERS database to characterize the safety profile of cladribine in treating MS. Lymphocytopenia emerged as the most frequently reported hematologic AE. As a purine analog, cladribine resists adenosine deaminase-mediated degradation, leading to significant accumulation in lymphocytes [16]. This accumulation disrupts DNA replication and repair, selectively inducing lymphocyte apoptosis [17]. Previous randomized controlled trials have confirmed lymphopenia as a known toxicity of cladribine [18], thereby supporting the validity of the results. Furthermore, studies have demonstrated that cladribine causes a dose-dependent reduction in key lymphocyte subsets, including CD4 + and CD8 + T cells [19]. Effective disease-modifying therapies (DMTs) often deplete memory B cells, and the extent of this depletion correlates with treatment efficacy [20].

Leukopenia and thrombocytopenia also emerged as significant AE signals. A prospective study found that leukocyte levels can remain reduced for up to 52 weeks, with neutrophils decreasing as early as week 1 and basophils by week 26 [21]. In phase III trials, thrombocytopenia was generally mild and uncommon, though severe cases were rare [10]. However, one case reported late-onset severe thrombocytopenia over a year post-treatment [22], suggesting a drug-induced autoimmune response rather than conventional hematopoietic suppression.

Pneumonia was the most common infection-related AE associated with cladribine. Although severe Lymphopenia (ALC < 0.5 × 10^9 cells/L) may increase pneumonia incidence, no significant difference in severity compared to the general population was observed [23]. This may be due to cladribine’s unique immunomodulatory mechanism, which selectively depletes CD19⁺ B cells and CD4⁺/CD8⁺ T cells [24], promoting adaptive immune system reconstitution and maintaining immune balance [3]. The clinical relevance of this safety feature was highlighted during the COVID-19 pandemic, where cladribine was sometimes prioritized for its balance of efficacy and safety [25].

The results of this study suggest that cladribine therapy is linked to a significant risk signal for drug-induced liver injury, a finding that aligns with data from the European spontaneous reporting database [9]. However, this stands in contrast to the low hepatotoxicity risk reported in early phase III clinical trials [18, 26]. This discrepancy may stem from the metabolic properties of cladribine, which is primarily excreted via the kidneys [27], as well as the limited sample size and relatively short observation periods in clinical trials.

The teratogenic potential of cladribine during pregnancy is underscored by its black box warning. This study has identified notable signals associated with preterm birth and pregnancy complications. It is important to note, however, that the majority of AE signals were derived from consumer spontaneous reports, which may be susceptible to misclassification of non-medical events or confounding factors. Therefore, positive signals at the SOC level should be interpreted cautiously. A prospective study reported one case of major congenital malformation in the cladribine-exposed group; however, no significant differences in overall pregnancy outcomes were observed compared to the unexposed group, potentially due to the limited sample size or relatively short follow-up period [28]. Animal studies have indicated that cladribine does not significantly affect female fertility but can cause reversible damage to male germ cells in mice, as well as demonstrating teratogenic effects in embryonic development studies in mice and rabbits [29]. Further large-scale studies with long-term follow-up are necessary to comprehensively evaluate the safety of cladribine use during pregnancy.

Several unexpected signals related to cladribine treatment were identified, including rheumatoid arthritis, polyarthritis, decreased blood potassium, hypothyroidism, eye hemorrhage, uveitis, retinal detachment, unresponsiveness to stimuli, brain edema, acute myocardial infarction, and completed suicide. The potential mechanism for rheumatoid arthritis and polyarthritis may involve an immune system imbalance caused by lymphocytopenia. It is noteworthy that cladribine has been previously investigated for the treatment of rheumatoid arthritis [30]. A preliminary study on refractory rheumatoid arthritis found that low-dose cladribine (0.05 mg/kg/week) selectively reduced T and B cell counts while maintaining normal levels of natural killer (NK) cells, effectively alleviating joint symptoms [31]. However, given that higher doses may cause extensive lymphocyte depletion, disrupt immune balance, and trigger the release of pro-inflammatory factors, we speculate that this could be a potential mechanism underlying joint-related adverse reactions. This suggests that the immunomodulatory effect of cladribine is highly dose-dependent, and its clinical application requires careful control of the dose threshold.

Additionally, the mechanisms behind some unexpected events remain unclear. For instance, decreased blood potassium may be related to electrolyte imbalances caused by drug-induced vomiting, while increased risks of hypothyroidism and acute myocardial infarction may be associated with autoimmune abnormalities and disease progression in MS [10, 32]. Ocular lesions, such as uveitis and retinal detachment, may arise from the direct involvement of the afferent visual and oculomotor systems in MS, or from neuroinflammatory injury to the optic nerve [33]. The risk of brain edema is closely linked to disruption of the blood–brain barrier due to early inflammation in MS [32, 34]. Furthermore, a Canadian cohort study revealed that the suicide rate among MS patients was 7.5 times higher than that of the general population, highlighting the need for enhanced mental health assessment during treatment [35]. For events like unresponsiveness to stimuli, no clear mechanism or literature support has been identified, warranting further investigation.

Gender-based subgroup analysis revealed distinct safety profiles. Females exhibited a higher susceptibility to alopecia and migraine. The significantly higher reporting of alopecia likely reflects greater psychosocial distress and reporting behavior among female patients [36]. Migraine, a common comorbidity in MS, can also be induced or exacerbated by DMTs [37]. In contrast, male patients had a higher risk of gait disturbance and sepsis. While the exact mechanism is not yet fully understood, this difference may be closely linked to the faster disease progression and more severe clinical deterioration observed in male patients [38–41].

Based on an age-stratified analysis, it was found that patients over 65 years old were more prone to leukopenia and UTIs. The heightened risk of leukopenia can be attributed to age-related reductions in the renal clearance of cladribine and compromised hematopoietic stem cell function [42–44]. Simultaneously, the increased risk of UTIs is consistent with studies showing that older MS patients undergoing high-efficacy treatments experience a significantly higher incidence of UTIs compared to their younger counterparts, likely attributable to comorbidities, iatrogenic factors, and possible drug-induced damage to the urothelial barrier [45, 46]. In contrast, younger adults (aged 18–64.9 years) most commonly reported nausea, a prevalent and generally mild adverse reaction [47].

### Analysis of the onset time of AEs cladribine

The data reveal that following cladribine treatment, the median time to the onset of AEs was 152 days, with a significant peak occurring in the first month of treatment (accounting for 28.18% of cases). Weibull distribution analysis yielded a shape parameter of 0.72 (95% CI 0.68–0.75), confirming the early clustering of AEs. The occurrence of acute toxicity early in the treatment course underscores the importance of initiating AE monitoring from the onset of therapy.

### Comparison with fingolimod

A within-indication comparison between cladribine and fingolimod highlights their distinct safety profiles. Fingolimod exhibited significantly stronger associations with macular edema and skin hemangioma, aligning with its mechanism of action [48]. As an S1P receptor modulator, fingolimod disrupts S1P signaling at the blood-retinal barrier, potentially leading to macular edema [1]. Regarding skin hemangioma, S1P plays a crucial role in regulating angiogenesis, and fingolimod’s modulation of this pathway may contribute to the development of such vascular lesions [49]. Additionally, its immunosuppressive effects are known to elevate the risk of cutaneous malignancies [50]. These findings emphasize the necessity for routine ophthalmological and dermatological monitoring in patients receiving fingolimod.

## Limitations

Several limitations characterize this study. Firstly, the FAERS is a passive monitoring system that relies on spontaneous reporting, which may introduce reporting biases and result in incomplete demographic data (e.g., gender and age). Secondly, the database lacks control for critical confounding factors, such as underlying conditions and concomitant medications. Consequently, these disproportionality findings suggest a statistical association but cannot be used to determine incidence or establish causation. Given these constraints, future prospective studies are necessary to confirm these potential associations.

## Conclusion

This study employed a systematic pharmacovigilance analysis using the FAERS database to assess the safety profile of cladribine in the treatment of MS. We identified common AEs, including lymphopenia, leukopenia, and thrombocytopenia, as well as potential new risk signals, such as rheumatoid arthritis, hypothyroidism, eye hemorrhage, uveitis, retinal detachment, brain edema, acute myocardial infarction, and completed suicide. Notable differences in the reporting proportion of certain AEs were observed across gender and age subgroups. Our research has deepened the understanding of cladribine’s safety profile. However, the newly identified risk signals warrant further investigation to elucidate their underlying mechanisms.

## Supplementary Information

Below is the link to the electronic supplementary material.

Supplementary file1 (DOCX 76 KB)

Supplementary file2 (DOCX 20 KB)

## Data Availability

All the data used in this study were obtained from the public FDA Adverse Event Reporting System (FAERS) database ( https:/www.fda.gov/drugs/drug-approvals-and-databases/fda-adverse-event-reporting-system-faers-database).
