# Supplementary material for: Post-marketing safety profile of cladribine in multiple sclerosis: a disproportionality analysis based on the FDA adverse event reporting system
Source: Int J Clin Pharm. 2025 Nov 15;48(2):623–35. doi: 10.1007/s11096-025-02041-8 (PMC12992368; doi:10.1007/s11096-025-02041-8)
Supplement: Supplementary file 1 — Supplementary file1 (DOCX 76 KB) [file 11096_2025_2041_MOESM1_ESM.docx]

| **Supplementary Table 1. ROR, PRR, BCPNN, and EBGM methods, formulas, and thresholds.** | | |
| --- | --- | --- |
| Method | Formula | Threshold |
| ROR | $ROR=\frac{a/c}{b/d}$ | $a\geq3$  ROR$\geq3$  95%CI(lower limit) > 1 |
|  | $SE(lnROR)=\sqrt{\frac{1}{a}+\frac{1}{b}+\frac{1}{c}+\frac{1}{d}}$ |  |
|  | $95\%CI=e^{ln(ROR)\pm1.96se}$ |  |
| PRR | $PRR=\frac{a/{(a+b)}}{c/{c+d}}$ | $a\geq3$  ROR$\geq2$  95%CI(lower limit) > 1 |
|  | $\mathrm{SE}\left( \mathrm{lnPRR} \right)=\frac{1}{a}-\frac{1}{a+b}+\frac{1}{c}-\frac{1}{c+d}$ |  |
|  | $95\%CI=e^{ln(PRR)\pm1.96se}$ |  |
| BCPNN | $IC=\log_{2}\frac{p(x,y)}{p(x)p(\gamma)}=\log_{2}\frac{a(a+b+c+d)}{(a+b)(a+c)}$ | IC025>0 |
|  | $E(IC)=\log_{2}\frac{(a+\gamma^{11})(a+b+c+d+a)(a+b+c+d+\beta)}{(a+b+c+d+\gamma)(a+b+\alpha1)(a+c+\beta1)}$ |  |
|  | $V\left( \mathrm{IC} \right)=\frac{1}{{(\ln2)}^{2}}[\frac{\left( a+b+c+d \right)-a+\gamma-\gamma^{11}}{\left( a+\gamma^{11} \right)}+\frac{\left( a+b+c+d \right)-\left( a+b \right)-\alpha1}{\left( a+b+\alpha1 \right)\left( a+b+c+d+\alpha\right)}+\frac{\left( a+b+c+d+a \right)-\left( a+c \right)+\beta-\beta1}{\left( a+b+\beta1 \right)\left( 1+a+b+c+d+\beta\right)}$ |  |
|  | $\gamma=\gamma^{11}\frac{\left( a+b+c+d+a \right)\left( a+b+c+d+\beta\right)}{\left( a+b+\alpha1 \right)\left( a+c+\beta1 \right)}$ |  |
|  | $IC-2SD=E\left( \mathrm{IC} \right)-2\sqrt{V\left( \mathrm{IC} \right)}$ |  |
| EBGM | $EBGM=\frac{a\left( a+b+c+d \right)}{\left( a+b \right)\left( a+c \right)}$ | EBGM05>2 |
|  | $\mathrm{SE}\left( \ln\mathrm{EBGM} \right)=\sqrt{\frac{1}{a}+\frac{1}{b}+\frac{1}{c}+\frac{1}{d}}$ |  |
|  | $95\%CI=e^{\ln\left( \mathrm{EBGM} \right)\pm1.96se}$ |  |

#### Abbreviations: ROR, Reporting Odds Ratio; PRR, Proportional Reporting Ratio; χ², Chi-square test value; EBGM, Empirical Bayesian Geometric Mean; EBGM05, the lower limit of the 95% confidence interval of EBGM; IC, Information Component; IC025, the lower limit of the 95% confidence interval of IC; CI, Confidence Interval.

**Supplementary Table 2. 2 × 2 fourfold table of disproportionality method.**

|  | Cladribine-related AEs | Non-cladribine-related AEs | Total |
| --- | --- | --- | --- |
| Cladribine | a | b | a + b |
| Non-cladribine | c | d | c + d |
| Total | a + c | b + d | N = a + b + c + d |

Footnotes: AE, adverse events; a, the number of cases with cladribine-associated target AE; b, the number of cases with cladribine-associated non-target AE; c, the number of cases with target AE of all other drugs; d, the number of cases with non-target AE of all other drugs.

**Supplementary Table 3. Signal strength of AEs at the SOC level for cladribine based on the FAERS database.**

| **SOC** | **Case reports** | **ROR (95%Cl)** | **PRR(χ²)** | **EBGM(EBGM05)** | **IC(IC025)** |
| --- | --- | --- | --- | --- | --- |
| Nervous system disorders | 2089 | 0.81 (0.77 - 0.85) | 0.84 (76.77) | 0.84 (0.81) | -0.25 (-0.32) |
| Infections and infestations | 1719 | 1.53 (1.45 - 1.61) | 1.46 (267.95) | 1.45 (1.39) | 0.54 (0.46) |
| General disorders and administration site conditions | 1706 | 0.68 (0.64 - 0.71) | 0.72 (228.14) | 0.72 (0.69) | -0.47 (-0.55) |
| Investigations | 1518 | 1.94 (1.83 - 2.04) | 1.83 (592.26) | 1.81 (1.73) | 0.85 (0.77) |
| Gastrointestinal disorders | 969 | 1.12 (1.05 - 1.20) | 1.11 (11.25) | 1.11 (1.05) | 0.15 (0.05) |
| Musculoskeletal and connective tissue disorders | 684 | 0.80 (0.74 - 0.87) | 0.81 (31.04) | 0.82 (0.76) | -0.29 (-0.41) |
| Injury, poisoning and procedural complications | 643 | 0.59 (0.55 - 0.64) | 0.61 (172.11) | 0.61 (0.57) | -0.70 (-0.82) |
| Skin and subcutaneous tissue disorders | 424 | 0.82 (0.74 - 0.90) | 0.83 (16.10) | 0.83 (0.76) | -0.27 (-0.42) |
| Respiratory, thoracic and mediastinal disorders | 395 | 0.89 (0.80 - 0.98) | 0.89 (5.50) | 0.89 (0.82) | -0.17 (-0.31) |
| Psychiatric disorders | 379 | 0.62 (0.56 - 0.68) | 0.63 (86.16) | 0.63 (0.58) | -0.66 (-0.81) |
| Neoplasms benign, malignant and unspecified (incl cysts and polyps) | 341 | 1.68 (1.51 - 1.88) | 1.67 (89.74) | 1.65 (1.51) | 0.72 (0.56) |
| Blood and lymphatic system disorders | 282 | 2.45 (2.18 - 2.77) | 2.42 (229.10) | 2.37 (2.14) | 1.25 (1.07) |
| Cardiac disorders | 264 | 1.64 (1.45 - 1.85) | 1.62 (62.44) | 1.61 (1.45) | 0.69 (0.50) |
| Eye disorders | 241 | 0.78 (0.69 - 0.89) | 0.78 (14.40) | 0.79 (0.71) | -0.35 (-0.53) |
| Metabolism and nutrition disorders | 225 | 1.56 (1.37 - 1.79) | 1.56 (44.01) | 1.54 (1.38) | 0.62 (0.43) |
| Renal and urinary disorders | 223 | 1.09 (0.95 - 1.24) | 1.08 (1.44) | 1.08 (0.97) | 0.11 (-0.08) |
| Surgical and medical procedures | 222 | 2.11 (1.85 - 2.42) | 2.09 (123.86) | 2.06 (1.84) | 1.04 (0.84) |
| Pregnancy, puerperium and perinatal conditions | 181 | 2.78 (2.39 - 3.23) | 2.75 (194.88) | 2.68 (2.37) | 1.42 (1.20) |
| Vascular disorders | 179 | 0.57 (0.49 - 0.66) | 0.57 (58.54) | 0.57 (0.51) | -0.80 (-1.02) |
| Hepatobiliary disorders | 141 | 2.17 (1.83 - 2.57) | 2.15 (84.86) | 2.12 (1.84) | 1.08 (0.84) |
| Reproductive system and breast disorders | 139 | 2.24 (1.89 - 2.66) | 2.23 (91.49) | 2.19 (1.90) | 1.13 (0.88) |
| Immune system disorders | 102 | 0.74 (0.61 - 0.90) | 0.74 (9.08) | 0.75 (0.63) | -0.42 (-0.71) |
| Ear and labyrinth disorders | 94 | 1.14 (0.93 - 1.40) | 1.14 (1.53) | 1.13 (0.96) | 0.18 (-0.12) |
| Endocrine disorders | 91 | 3.14 (2.54 - 3.87) | 3.12 (125.44) | 3.02 (2.53) | 1.60 (1.29) |
| Social circumstances | 41 | 0.93 (0.68 - 1.26) | 0.93 (0.24) | 0.93 (0.72) | -0.11 (-0.56) |
| Product issues | 16 | 0.27 (0.17 - 0.45) | 0.27 (30.72) | 0.28 (0.18) | -1.85 (-2.55) |
| Congenital, familial and genetic disorders | 7 | 0.64 (0.31 - 1.36) | 0.64 (1.36) | 0.65 (0.35) | -0.62 (-1.65) |

Abbreviations: AEs, Adverse Events; SOC, System Organ Class. ROR, Reporting Odds Ratio; PRR, Proportional Reporting Ratio; χ², Chi-square test value; EBGM, Empirical Bayesian Geometric Mean; EBGM05, the lower limit of the 95% confidence interval of EBGM; IC, Information Component; IC025, the lower limit of the 95% confidence interval of IC; CI, Confidence Interval.

**Supplementary Table 4. Positive preferred terms (PT) of cladribine meeting the criteria of four disproportionality methods.**

| **PT** | **Case reports** | **ROR (95%Cl)** | **PRR(χ²)** | **EBGM(EBGM05)** | **IC(IC025)** |
| --- | --- | --- | --- | --- | --- |
| Lymphocyte count decreased | 336 | 6.34 (5.66 - 7.10) | 6.21 (1345.73) | 5.75 (5.23) | 2.52 (2.36) |
| White blood cell count decreased | 236 | 3.82 (3.35 - 4.36) | 3.77 (456.64) | 3.62 (3.24) | 1.86 (1.66) |
| Pneumonia | 190 | 2.85 (2.46 - 3.30) | 2.83 (215.87) | 2.75 (2.43) | 1.46 (1.24) |
| Lymphopenia | 111 | 4.19 (3.45 - 5.08) | 4.16 (250.99) | 3.97 (3.38) | 1.99 (1.71) |
| Lower respiratory tract infection | 71 | 6.58 (5.15 - 8.41) | 6.55 (303.64) | 6.04 (4.92) | 2.60 (2.24) |
| Alanine aminotransferase increased | 60 | 3.61 (2.78 - 4.69) | 3.60 (106.83) | 3.46 (2.78) | 1.79 (1.41) |
| Aspartate aminotransferase increased | 48 | 4.60 (3.43 - 6.16) | 4.59 (125.79) | 4.35 (3.40) | 2.12 (1.69) |
| Nephrolithiasis | 48 | 2.64 (1.98 - 3.52) | 2.63 (46.72) | 2.57 (2.02) | 1.36 (0.94) |
| Kidney infection | 39 | 4.08 (2.95 - 5.64) | 4.07 (85.15) | 3.89 (2.97) | 1.96 (1.49) |
| Platelet count decreased | 37 | 3.84 (2.76 - 5.35) | 3.83 (73.22) | 3.68 (2.78) | 1.88 (1.40) |
| Hypothyroidism | 32 | 5.04 (3.52 - 7.23) | 5.03 (96.03) | 4.74 (3.51) | 2.25 (1.72) |
| Leukopenia | 32 | 2.89 (2.03 - 4.12) | 2.88 (37.72) | 2.80 (2.08) | 1.49 (0.97) |
| Neutrophil count decreased | 31 | 6.13 (4.24 - 8.87) | 6.12 (121.42) | 5.68 (4.17) | 2.51 (1.97) |
| Diverticulitis | 30 | 5.55 (3.82 - 8.06) | 5.54 (102.79) | 5.18 (3.79) | 2.37 (1.83) |
| Drug-induced liver injury | 22 | 5.94 (3.84 - 9.19) | 5.93 (82.65) | 5.52 (3.83) | 2.46 (1.84) |
| Haematochezia | 22 | 5.09 (3.30 - 7.86) | 5.09 (66.99) | 4.79 (3.33) | 2.26 (1.64) |
| Rheumatoid arthritis | 21 | 5.64 (3.61 - 8.82) | 5.64 (73.72) | 5.27 (3.63) | 2.40 (1.76) |
| Hypophagia | 19 | 5.80 (3.63 - 9.28) | 5.79 (69.22) | 5.40 (3.65) | 2.43 (1.76) |
| Thrombocytopenia | 19 | 3.64 (2.30 - 5.79) | 3.64 (34.47) | 3.50 (2.38) | 1.81 (1.14) |
| Dysmenorrhoea | 17 | 13.82 (8.19 - 23.33) | 13.80 (166.53) | 11.56 (7.46) | 3.53 (2.79) |
| Blood potassium decreased | 17 | 3.15 (1.93 - 5.12) | 3.15 (23.75) | 3.05 (2.03) | 1.61 (0.91) |
| Cyst | 15 | 4.20 (2.49 - 7.09) | 4.20 (34.36) | 4.01 (2.59) | 2.00 (1.26) |
| Blood bilirubin increased | 14 | 4.03 (2.35 - 6.91) | 4.02 (29.97) | 3.85 (2.45) | 1.94 (1.18) |
| Autoimmune thyroiditis | 12 | 4.29 (2.39 - 7.69) | 4.28 (28.34) | 4.08 (2.50) | 2.03 (1.20) |
| Eye haemorrhage | 11 | 6.88 (3.69 - 12.80) | 6.87 (49.91) | 6.31 (3.75) | 2.66 (1.78) |
| Unresponsive to stimuli | 11 | 4.61 (2.50 - 8.51) | 4.61 (29.04) | 4.37 (2.62) | 2.13 (1.27) |
| Premature baby | 10 | 7.47 (3.88 - 14.38) | 7.47 (50.24) | 6.80 (3.93) | 2.77 (1.85) |
| Angioedema | 9 | 4.24 (2.16 - 8.32) | 4.24 (20.89) | 4.04 (2.30) | 2.01 (1.07) |
| Acute myocardial infarction | 9 | 3.70 (1.89 - 7.25) | 3.70 (16.78) | 3.55 (2.03) | 1.83 (0.89) |
| Complication of pregnancy | 8 | 16.25 (7.49 - 35.27) | 16.24 (91.53) | 13.19 (6.90) | 3.72 (2.66) |
| Graves' disease | 8 | 10.40 (4.93 - 21.94) | 10.39 (58.55) | 9.10 (4.87) | 3.19 (2.15) |
| Myelodysplastic syndrome | 8 | 8.81 (4.21 - 18.45) | 8.81 (48.76) | 7.88 (4.24) | 2.98 (1.95) |
| Brain oedema | 8 | 6.12 (2.96 - 12.63) | 6.11 (31.28) | 5.67 (3.09) | 2.50 (1.50) |
| Cervical dysplasia | 8 | 5.00 (2.43 - 10.26) | 5.00 (23.75) | 4.71 (2.58) | 2.24 (1.24) |
| Uveitis | 8 | 4.86 (2.37 - 9.97) | 4.86 (22.8) | 4.59 (2.51) | 2.20 (1.20) |
| Faeces discoloured | 8 | 4.77 (2.33 - 9.78) | 4.77 (22.19) | 4.51 (2.47) | 2.17 (1.18) |
| Pneumonia viral | 7 | 12.64 (5.62 - 28.40) | 12.63 (62.77) | 10.74 (5.45) | 3.42 (2.32) |
| Completed suicide | 7 | 7.46 (3.41 - 16.31) | 7.45 (35.09) | 6.79 (3.53) | 2.76 (1.69) |
| Subcutaneous abscess | 7 | 6.23 (2.87 - 13.54) | 6.23 (28.04) | 5.77 (3.02) | 2.53 (1.46) |
| Pneumonia bacterial | 7 | 4.59 (2.13 - 9.89) | 4.59 (18.38) | 4.36 (2.29) | 2.12 (1.07) |
| Retinal detachment | 7 | 4.37 (2.03 - 9.40) | 4.37 (17.06) | 4.16 (2.19) | 2.06 (1.00) |
| Pertussis | 6 | 22.94 (9.04 - 58.18) | 22.93 (93.00) | 17.21 (7.90) | 4.10 (2.87) |
| Polyarthritis | 6 | 16.25 (6.64 - 39.75) | 16.24 (68.65) | 13.19 (6.24) | 3.72 (2.51) |
| Metastases to central nervous system | 6 | 9.28 (3.95 - 21.84) | 9.28 (38.79) | 8.24 (4.03) | 3.04 (1.88) |
| Blood creatine phosphokinase increased | 6 | 6.96 (3.00 - 16.16) | 6.96 (27.66) | 6.38 (3.16) | 2.67 (1.52) |
| Hepatitis c | 6 | 6.39 (2.76 - 14.79) | 6.39 (24.83) | 5.91 (2.93) | 2.56 (1.42) |
| Synovial cyst | 6 | 5.34 (2.32 - 12.28) | 5.34 (19.55) | 5.01 (2.50) | 2.32 (1.19) |
| Arthritis infective | 6 | 4.94 (2.15 - 11.32) | 4.93 (17.49) | 4.66 (2.32) | 2.22 (1.08) |
| Pericarditis | 6 | 4.75 (2.08 - 10.89) | 4.75 (16.57) | 4.50 (2.25) | 2.17 (1.04) |
| Plasma cell myeloma | 6 | 4.59 (2.00 - 10.5) | 4.59 (15.71) | 4.35 (2.17) | 2.12 (0.99) |
| Atypical pneumonia | 6 | 4.28 (1.88 - 9.79) | 4.28 (14.17) | 4.08 (2.04) | 2.03 (0.90) |
| Pancytopenia | 6 | 4.24 (1.86 - 9.68) | 4.24 (13.93) | 4.04 (2.02) | 2.01 (0.88) |
| Dyspnoea at rest | 5 | 24.99 (8.91 - 70.12) | 24.98 (83.14) | 18.32 (7.73) | 4.20 (2.84) |
| Hepatitis toxic | 5 | 14.13 (5.37 - 37.16) | 14.12 (50.08) | 11.78 (5.24) | 3.56 (2.26) |
| Postmenopausal haemorrhage | 5 | 12.50 (4.80 - 32.55) | 12.49 (44.34) | 10.64 (4.78) | 3.41 (2.13) |
| Atrial flutter | 5 | 12.03 (4.63 - 31.25) | 12.03 (42.66) | 10.31 (4.64) | 3.37 (2.08) |
| Low birth weight baby | 5 | 12.03 (4.63 - 31.25) | 12.03 (42.66) | 10.31 (4.64) | 3.37 (2.08) |
| Infected cyst | 5 | 10.83 (4.20 - 27.92) | 10.83 (38.22) | 9.42 (4.27) | 3.24 (1.96) |
| Fluid intake reduced | 5 | 6.02 (2.41 - 15.04) | 6.01 (19.13) | 5.59 (2.60) | 2.48 (1.25) |
| B-cell lymphoma | 5 | 5.91 (2.36 - 14.76) | 5.91 (18.68) | 5.50 (2.55) | 2.46 (1.22) |
| Cholecystitis infective | 5 | 5.60 (2.25 - 13.97) | 5.60 (17.39) | 5.23 (2.44) | 2.39 (1.16) |
| Mean cell volume increased | 5 | 5.08 (2.04 - 12.61) | 5.07 (15.18) | 4.78 (2.23) | 2.26 (1.03) |
| Nodule | 5 | 4.85 (1.95 - 12.03) | 4.85 (14.21) | 4.58 (2.14) | 2.20 (0.97) |
| Thyrotoxic crisis | 4 | 17.33 (5.75 - 52.21) | 17.32 (48.57) | 13.89 (5.52) | 3.80 (2.35) |
| Autoimmune thyroid disorder | 4 | 16.24 (5.43 - 48.60) | 16.24 (45.76) | 13.19 (5.27) | 3.72 (2.29) |
| Amyotrophic lateral sclerosis | 4 | 13.00 (4.44 - 38.03) | 12.99 (36.90) | 10.99 (4.48) | 3.46 (2.04) |
| Physical disability | 4 | 9.28 (3.26 - 26.47) | 9.28 (25.86) | 8.24 (3.43) | 3.04 (1.66) |
| Hypertransaminasaemia | 4 | 8.66 (3.05 - 24.59) | 8.66 (23.92) | 7.76 (3.24) | 2.96 (1.58) |
| Abdominal infection | 4 | 8.38 (2.96 - 23.75) | 8.38 (23.03) | 7.54 (3.15) | 2.91 (1.54) |
| Retinal tear | 4 | 8.12 (2.87 - 22.97) | 8.12 (22.20) | 7.33 (3.07) | 2.87 (1.50) |
| Escherichia sepsis | 4 | 7.64 (2.71 - 21.54) | 7.64 (20.66) | 6.94 (2.92) | 2.80 (1.42) |
| Hepatitis acute | 4 | 6.66 (2.38 - 18.65) | 6.66 (17.46) | 6.14 (2.59) | 2.62 (1.26) |
| Skin necrosis | 4 | 6.50 (2.32 - 18.16) | 6.50 (16.91) | 6.00 (2.54) | 2.58 (1.22) |
| Cardio-respiratory arrest | 4 | 5.91 (2.12 - 16.44) | 5.91 (14.94) | 5.50 (2.33) | 2.46 (1.10) |
| Lipoma | 4 | 5.78 (2.08 - 16.06) | 5.77 (14.50) | 5.38 (2.29) | 2.43 (1.08) |
| Tearfulness | 4 | 5.65 (2.03 - 15.70) | 5.65 (14.08) | 5.28 (2.24) | 2.40 (1.05) |
| Dyschezia | 4 | 5.41 (1.95 - 15.02) | 5.41 (13.29) | 5.07 (2.16) | 2.34 (0.99) |
| Genital neoplasm malignant female | 3 | 64.97 (13.11 - 321.95) | 64.96 (94.46) | 32.98 (8.64) | 5.04 (3.23) |
| Ovarian germ cell teratoma benign | 3 | 38.98 (9.32 - 163.14) | 38.97 (69.38) | 24.73 (7.47) | 4.63 (2.89) |
| Follicular thyroid cancer | 3 | 32.49 (8.12 - 129.91) | 32.48 (61.02) | 21.99 (6.89) | 4.46 (2.75) |
| Cutaneous vasculitis | 3 | 32.49 (8.12 - 129.91) | 32.48 (61.02) | 21.99 (6.89) | 4.46 (2.75) |
| Carotid arteriosclerosis | 3 | 32.49 (8.12 - 129.91) | 32.48 (61.02) | 21.99 (6.89) | 4.46 (2.75) |
| Pneumonia streptococcal | 3 | 24.36 (6.46 - 91.85) | 24.36 (48.87) | 17.99 (5.93) | 4.17 (2.50) |
| Cachexia | 3 | 21.66 (5.86 - 80.01) | 21.65 (44.32) | 16.49 (5.53) | 4.04 (2.39) |
| Ear haemorrhage | 3 | 21.66 (5.86 - 80.01) | 21.65 (44.32) | 16.49 (5.53) | 4.04 (2.39) |
| Otitis externa | 3 | 19.49 (5.36 - 70.83) | 19.49 (40.47) | 15.22 (5.17) | 3.93 (2.29) |
| Metastases to spine | 3 | 16.24 (4.58 - 57.57) | 16.24 (34.32) | 13.19 (4.58) | 3.72 (2.11) |
| Myelin oligodendrocyte glycoprotein antibody-associated disease | 3 | 16.24 (4.58 - 57.57) | 16.24 (34.32) | 13.19 (4.58) | 3.72 (2.11) |
| Bilirubin conjugated increased | 3 | 14.99 (4.27 - 52.62) | 14.99 (31.83) | 12.37 (4.33) | 3.63 (2.02) |
| Breast discharge | 3 | 13.92 (4.00 - 48.45) | 13.92 (29.63) | 11.64 (4.10) | 3.54 (1.95) |
| Cystocele | 3 | 13.92 (4.00 - 48.45) | 13.92 (29.63) | 11.64 (4.10) | 3.54 (1.95) |
| Myelosuppression | 3 | 12.99 (3.76 - 44.89) | 12.99 (27.67) | 10.99 (3.90) | 3.46 (1.87) |
| Toxic skin eruption | 3 | 12.18 (3.55 - 41.81) | 12.18 (25.92) | 10.41 (3.71) | 3.38 (1.80) |
| Lactic acidosis | 3 | 12.18 (3.55 - 41.81) | 12.18 (25.92) | 10.41 (3.71) | 3.38 (1.80) |
| Hepatic neoplasm | 3 | 12.18 (3.55 - 41.81) | 12.18 (25.92) | 10.41 (3.71) | 3.38 (1.80) |
| Uterine infection | 3 | 11.47 (3.36 - 39.13) | 11.46 (24.35) | 9.89 (3.54) | 3.31 (1.73) |
| Sinusitis bacterial | 3 | 10.83 (3.19 - 36.77) | 10.83 (22.93) | 9.42 (3.39) | 3.24 (1.67) |
| Wound infection staphylococcal | 3 | 9.75 (2.90 - 32.80) | 9.74 (20.47) | 8.60 (3.12) | 3.10 (1.55) |
| Blood urea decreased | 3 | 9.75 (2.90 - 32.80) | 9.74 (20.47) | 8.60 (3.12) | 3.10 (1.55) |
| Vulvovaginal burning sensation | 3 | 9.75 (2.90 - 32.80) | 9.74 (20.47) | 8.60 (3.12) | 3.10 (1.55) |
| Anembryonic gestation | 3 | 9.28 (2.77 - 31.12) | 9.28 (19.39) | 8.24 (3.00) | 3.04 (1.49) |
| Antinuclear antibody positive | 3 | 9.28 (2.77 - 31.12) | 9.28 (19.39) | 8.24 (3.00) | 3.04 (1.49) |
| Endometrial adenocarcinoma | 3 | 8.12 (2.45 - 26.97) | 8.12 (16.65) | 7.33 (2.68) | 2.87 (1.33) |
| Central nervous system infection | 3 | 7.80 (2.35 - 25.83) | 7.79 (15.87) | 7.07 (2.59) | 2.82 (1.28) |
| Urinary tract obstruction | 3 | 7.80 (2.35 - 25.83) | 7.79 (15.87) | 7.07 (2.59) | 2.82 (1.28) |
| Cardiac dysfunction | 3 | 7.80 (2.35 - 25.83) | 7.79 (15.87) | 7.07 (2.59) | 2.82 (1.28) |
| Vertebral foraminal stenosis | 3 | 7.50 (2.27 - 24.77) | 7.50 (15.14) | 6.82 (2.51) | 2.77 (1.23) |
| Tympanic membrane perforation | 3 | 7.22 (2.19 - 23.80) | 7.22 (14.46) | 6.60 (2.43) | 2.72 (1.19) |
| International normalised ratio increased | 3 | 7.22 (2.19 - 23.80) | 7.22 (14.46) | 6.60 (2.43) | 2.72 (1.19) |
| Sudden hearing loss | 3 | 6.96 (2.12 - 22.90) | 6.96 (13.83) | 6.38 (2.36) | 2.67 (1.14) |
| Blood immunoglobulin g increased | 3 | 6.50 (1.98 - 21.29) | 6.50 (12.68) | 6.00 (2.22) | 2.58 (1.06) |
| Urinary tract infection enterococcal | 3 | 5.91 (1.81 - 19.26) | 5.91 (11.21) | 5.50 (2.04) | 2.46 (0.94) |
| Invasive lobular breast carcinoma | 3 | 5.91 (1.81 - 19.26) | 5.91 (11.21) | 5.50 (2.04) | 2.46 (0.94) |

Abbreviations: PT, Preferred Terms; ROR, Reporting Odds Ratio; PRR, Proportional Reporting Ratio; χ², Chi-square test value; EBGM, Empirical Bayesian Geometric Mean; EBGM05, the lower limit of the 95% confidence interval of EBGM; IC, Information Component; IC025, the lower limit of the 95% confidence interval of IC; CI, Confidence Interval.

**Supplementary Table 5. Top 50 adverse events of cladribine in the FAERS database, categorized and ranked by SOC.**

| SOC | PT | Case reports | ROR (95%Cl) | PRR () | EBGM(EBGM05) | IC(IC025) |
| --- | --- | --- | --- | --- | --- | --- |
| Infections and infestations | Pneumonia | 190 | 2.85 (2.46 - 3.3) | 2.83 (215.87) | 2.75 (2.43) | 1.46 (1.24) |
|  | Lower respiratory tract infection | 71 | 6.58 (5.15 - 8.41) | 6.55 (303.64) | 6.04 (4.92) | 2.6 (2.24) |
|  | Kidney infection | 39 | 4.08 (2.95 - 5.64) | 4.07 (85.15) | 3.89 (2.97) | 1.96 (1.49) |
|  | Diverticulitis | 30 | 5.55 (3.82 - 8.06) | 5.54 (102.79) | 5.18 (3.79) | 2.37 (1.83) |
|  | Pneumonia viral | 7 | 12.64 (5.62 - 28.4) | 12.63 (62.77) | 10.74 (5.45) | 3.42 (2.32) |
|  | Subcutaneous abscess | 7 | 6.23 (2.87 - 13.54) | 6.23 (28.04) | 5.77 (3.02) | 2.53 (1.46) |
|  | Pneumonia bacterial | 7 | 4.59 (2.13 - 9.89) | 4.59 (18.38) | 4.36 (2.29) | 2.12 (1.07) |
|  | Pertussis | 6 | 22.94 (9.04 - 58.18) | 22.93 (93) | 17.21 (7.9) | 4.1 (2.87) |
|  | Hepatitis c | 6 | 6.39 (2.76 - 14.79) | 6.39 (24.83) | 5.91 (2.93) | 2.56 (1.42) |
|  | Arthritis infective | 6 | 4.94 (2.15 - 11.32) | 4.93 (17.49) | 4.66 (2.32) | 2.22 (1.08) |
| Investigations | Lymphocyte count decreased | 336 | 6.34 (5.66 - 7.1) | 6.21 (1345.73) | 5.75 (5.23) | 2.52 (2.36) |
|  | White blood cell count decreased | 236 | 3.82 (3.35 - 4.36) | 3.77 (456.64) | 3.62 (3.24) | 1.86 (1.66) |
|  | Alanine aminotransferase increased | 60 | 3.61 (2.78 - 4.69) | 3.6 (106.83) | 3.46 (2.78) | 1.79 (1.41) |
|  | Aspartate aminotransferase increased | 48 | 4.6 (3.43 - 6.16) | 4.59 (125.79) | 4.35 (3.4) | 2.12 (1.69) |
|  | Platelet count decreased | 37 | 3.84 (2.76 - 5.35) | 3.83 (73.22) | 3.68 (2.78) | 1.88 (1.4) |
|  | Neutrophil count decreased | 31 | 6.13 (4.24 - 8.87) | 6.12 (121.42) | 5.68 (4.17) | 2.51 (1.97) |
|  | Blood potassium decreased | 17 | 3.15 (1.93 - 5.12) | 3.15 (23.75) | 3.05 (2.03) | 1.61 (0.91) |
|  | Blood bilirubin increased | 14 | 4.03 (2.35 - 6.91) | 4.02 (29.97) | 3.85 (2.45) | 1.94 (1.18) |
|  | Blood creatine phosphokinase increased | 6 | 6.96 (3 - 16.16) | 6.96 (27.66) | 6.38 (3.16) | 2.67 (1.52) |
| Blood and lymphatic system disorders | Lymphopenia | 111 | 4.19 (3.45 - 5.08) | 4.16 (250.99) | 3.97 (3.38) | 1.99 (1.71) |
|  | Leukopenia | 32 | 2.89 (2.03 - 4.12) | 2.88 (37.72) | 2.8 (2.08) | 1.49 (0.97) |
|  | Thrombocytopenia | 19 | 3.64 (2.3 - 5.79) | 3.64 (34.47) | 3.5 (2.38) | 1.81 (1.14) |
| Endocrine disorders | Hypothyroidism | 32 | 5.04 (3.52 - 7.23) | 5.03 (96.03) | 4.74 (3.51) | 2.25 (1.72) |
|  | Autoimmune thyroiditis | 12 | 4.29 (2.39 - 7.69) | 4.28 (28.34) | 4.08 (2.5) | 2.03 (1.2) |
|  | Graves' disease | 8 | 10.4 (4.93 - 21.94) | 10.39 (58.55) | 9.1 (4.87) | 3.19 (2.15) |
| Eye disorders | Eye haemorrhage | 11 | 6.88 (3.69 - 12.8) | 6.87 (49.91) | 6.31 (3.75) | 2.66 (1.78) |
|  | Uveitis | 8 | 4.86 (2.37 - 9.97) | 4.86 (22.8) | 4.59 (2.51) | 2.2 (1.2) |
|  | Retinal detachment | 7 | 4.37 (2.03 - 9.4) | 4.37 (17.06) | 4.16 (2.19) | 2.06 (1) |
| Musculoskeletal and connective tissue disorders | Rheumatoid arthritis | 21 | 5.64 (3.61 - 8.82) | 5.64 (73.72) | 5.27 (3.63) | 2.4 (1.76) |
|  | Polyarthritis | 6 | 16.25 (6.64 - 39.75) | 16.24 (68.65) | 13.19 (6.24) | 3.72 (2.51) |
|  | Synovial cyst | 6 | 5.34 (2.32 - 12.28) | 5.34 (19.55) | 5.01 (2.5) | 2.32 (1.19) |
| Neoplasms benign, malignant and unspecified | Myelodysplastic syndrome | 8 | 8.81 (4.21 - 18.45) | 8.81 (48.76) | 7.88 (4.24) | 2.98 (1.95) |
|  | Metastases to central nervous system | 6 | 9.28 (3.95 - 21.84) | 9.28 (38.79) | 8.24 (4.03) | 3.04 (1.88) |
|  | Plasma cell myeloma | 6 | 4.59 (2 - 10.5) | 4.59 (15.71) | 4.35 (2.17) | 2.12 (0.99) |
| Cardiac disorders | Acute myocardial infarction | 9 | 3.7 (1.89 - 7.25) | 3.7 (16.78) | 3.55 (2.03) | 1.83 (0.89) |
|  | Pericarditis | 6 | 4.75 (2.08 - 10.89) | 4.75 (16.57) | 4.5 (2.25) | 2.17 (1.04) |
| Gastrointestinal disorders | Haematochezia | 22 | 5.09 (3.3 - 7.86) | 5.09 (66.99) | 4.79 (3.33) | 2.26 (1.64) |
|  | Faeces discoloured | 8 | 4.77 (2.33 - 9.78) | 4.77 (22.19) | 4.51 (2.47) | 2.17 (1.18) |
| Nervous system disorders | Unresponsive to stimuli | 11 | 4.61 (2.5 - 8.51) | 4.61 (29.04) | 4.37 (2.62) | 2.13 (1.27) |
|  | Brain oedema | 8 | 6.12 (2.96 - 12.63) | 6.11 (31.28) | 5.67 (3.09) | 2.5 (1.5) |
| Pregnancy, puerperium and perinatal conditions | Premature baby | 10 | 7.47 (3.88 - 14.38) | 7.47 (50.24) | 6.8 (3.93) | 2.77 (1.85) |
|  | Complication of pregnancy | 8 | 16.25 (7.49 - 35.27) | 16.24 (91.53) | 13.19 (6.9) | 3.72 (2.66) |
| Reproductive system and breast disorders | Dysmenorrhoea | 17 | 13.82 (8.19 - 23.33) | 13.8 (166.53) | 11.56 (7.46) | 3.53 (2.79) |
|  | Cervical dysplasia | 8 | 5 (2.43 - 10.26) | 5 (23.75) | 4.71 (2.58) | 2.24 (1.24) |
| Renal and urinary disorders | Nephrolithiasis | 48 | 2.64 (1.98 - 3.52) | 2.63 (46.72) | 2.57 (2.02) | 1.36 (0.94) |
| Hepatobiliary disorders | Drug-induced liver injury | 22 | 5.94 (3.84 - 9.19) | 5.93 (82.65) | 5.52 (3.83) | 2.46 (1.84) |
| Metabolism and nutrition disorders | Hypophagia | 19 | 5.8 (3.63 - 9.28) | 5.79 (69.22) | 5.4 (3.65) | 2.43 (1.76) |
| General disorders and administration site conditions | Cyst | 15 | 4.2 (2.49 - 7.09) | 4.2 (34.36) | 4.01 (2.59) | 2 (1.26) |
| Skin and subcutaneous tissue disorders | Angioedema | 9 | 4.24 (2.16 - 8.32) | 4.24 (20.89) | 4.04 (2.3) | 2.01 (1.07) |
| Psychiatric disorders | Completed suicide | 7 | 7.46 (3.41 - 16.31) | 7.45 (35.09) | 6.79 (3.53) | 2.76 (1.69) |

Footnotes: PT, preferred term; SOC, system organ classification. CI, confidence interval; EBGM, empirical bayesian geometric mean; EBGM05, the lower limit of 95% confidence interval of EBGM; IC, information component; IC025, the lower limit of 95% confidence interval of the IC; PRR, proportional reporting ratio; PT, preferred term; ROR, reporting odds ratio; χ^2^, Chi-squared.

**Supplementary Table 6. Top 50 adverse events of fingolimod in the FAERS database, categorized and ranked by SOC.**

| **SOC** | **PT** | **Case reports** | **ROR (95%Cl)** | **PRR(χ²)** | **EBGM(EBGM05)** | **IC(IC025)** |
| --- | --- | --- | --- | --- | --- | --- |
| Investigations | white blood cell count decreased | 1786 | 5.33 (5.01 - 5.66) | 5.25 (3582.95) | 3.47 (3.29) | 1.79 (1.71) |
|  | lymphocyte count decreased | 1498 | 4.79 (4.48 - 5.11) | 4.73 (2680.52) | 3.26 (3.09) | 1.7 (1.62) |
|  | heart rate decreased | 621 | 7.52 (6.72 - 8.41) | 7.48 (1717.71) | 4.19 (3.81) | 2.07 (1.92) |
|  | hepatic enzyme increased | 621 | 5.03 (4.54 - 5.57) | 5.01 (1179.98) | 3.37 (3.09) | 1.75 (1.62) |
|  | alanine aminotransferase increased | 549 | 6.96 (6.19 - 7.83) | 6.93 (1426.63) | 4.03 (3.66) | 2.01 (1.86) |
|  | aspartate aminotransferase increased | 321 | 5.89 (5.08 - 6.83) | 5.88 (717.93) | 3.69 (3.26) | 1.88 (1.69) |
|  | gamma-glutamyltransferase increased | 317 | 7.2 (6.16 - 8.41) | 7.18 (847.84) | 4.1 (3.6) | 2.04 (1.84) |
|  | blood alkaline phosphatase increased | 170 | 8.75 (7 - 10.94) | 8.74 (528.47) | 4.51 (3.74) | 2.17 (1.9) |
|  | blood bilirubin increased | 105 | 5.86 (4.53 - 7.58) | 5.85 (233.89) | 3.69 (2.97) | 1.88 (1.54) |
|  | low density lipoprotein increased | 93 | 12.05 (8.65 - 16.79) | 12.04 (353.83) | 5.15 (3.9) | 2.36 (1.98) |
|  | lymphocyte count increased | 79 | 5.91 (4.39 - 7.95) | 5.9 (177.38) | 3.7 (2.89) | 1.89 (1.5) |
|  | high density lipoprotein increased | 51 | 30.82 (16.43 - 57.81) | 30.81 (280.18) | 6.68 (3.95) | 2.74 (2.2) |
|  | hepatic enzyme abnormal | 44 | 4.2 (2.9 - 6.09) | 4.2 (67.86) | 3.02 (2.22) | 1.6 (1.09) |
|  | blood alkaline phosphatase decreased | 38 | 13.12 (7.7 - 22.36) | 13.12 (151.4) | 5.31 (3.4) | 2.41 (1.82) |
| Nervous system disorders | central nervous system lesion | 855 | 4.59 (4.21 - 5) | 4.56 (1461.4) | 3.18 (2.96) | 1.67 (1.55) |
|  | movement disorder | 383 | 2.95 (2.62 - 3.32) | 2.94 (349.57) | 2.38 (2.16) | 1.25 (1.08) |
|  | ataxia | 140 | 2.92 (2.4 - 3.55) | 2.92 (125.78) | 2.37 (2.01) | 1.24 (0.97) |
|  | dizziness postural | 69 | 4.31 (3.2 - 5.81) | 4.31 (110.08) | 3.08 (2.4) | 1.62 (1.22) |
|  | paresis | 43 | 4.39 (3.01 - 6.41) | 4.39 (70.12) | 3.11 (2.27) | 1.64 (1.13) |
|  | dysaesthesia | 39 | 3.87 (2.63 - 5.71) | 3.87 (54.17) | 2.87 (2.07) | 1.52 (0.99) |
| Eye disorders | vision blurred | 856 | 2.94 (2.72 - 3.19) | 2.93 (775.6) | 2.37 (2.22) | 1.25 (1.13) |
|  | eye pain | 338 | 3.45 (3.03 - 3.93) | 3.45 (398.24) | 2.66 (2.38) | 1.41 (1.23) |
|  | macular oedema | 214 | 15.86 (12.49 - 20.14) | 15.83 (934.15) | 5.66 (4.63) | 2.5 (2.24) |
|  | photophobia | 103 | 3.51 (2.77 - 4.44) | 3.51 (124.36) | 2.69 (2.21) | 1.43 (1.1) |
|  | ocular discomfort | 76 | 3.83 (2.9 - 5.05) | 3.83 (103.85) | 2.85 (2.26) | 1.51 (1.13) |
|  | vitreous floaters | 59 | 3.85 (2.81 - 5.29) | 3.85 (81.4) | 2.86 (2.2) | 1.52 (1.08) |
|  | asthenopia | 50 | 4.07 (2.88 - 5.76) | 4.07 (74.22) | 2.97 (2.22) | 1.57 (1.1) |
|  | optic atrophy | 34 | 5.6 (3.58 - 8.77) | 5.6 (72.5) | 3.6 (2.47) | 1.85 (1.26) |
| Neoplasms benign, malignant and unspecified (incl cysts and polyps) | melanocytic naevus | 252 | 18.49 (14.66 - 23.34) | 18.45 (1173.66) | 5.92 (4.87) | 2.57 (2.33) |
|  | seborrhoeic keratosis | 123 | 44.63 (27.82 - 71.6) | 44.58 (733.01) | 7.1 (4.78) | 2.83 (2.47) |
|  | skin papilloma | 59 | 8.39 (5.77 - 12.21) | 8.39 (177.97) | 4.42 (3.23) | 2.15 (1.68) |
|  | haemangioma of skin | 55 | 56.98 (25.95 - 125.13) | 56.96 (341.39) | 7.32 (3.79) | 2.87 (2.34) |
|  | squamous cell carcinoma | 50 | 3.45 (2.47 - 4.84) | 3.45 (59) | 2.66 (2.01) | 1.41 (0.95) |
|  | fibrous histiocytoma | 38 | 39.36 (17.58 - 88.15) | 39.35 (220.95) | 6.97 (3.55) | 2.8 (2.17) |
| Cardiac disorders | bradycardia | 296 | 4.27 (3.7 - 4.93) | 4.26 (465.17) | 3.05 (2.71) | 1.61 (1.41) |
|  | sinus bradycardia | 91 | 11.79 (8.45 - 16.45) | 11.78 (342.02) | 5.11 (3.86) | 2.35 (1.97) |
|  | atrioventricular block first degree | 86 | 20.13 (13.35 - 30.35) | 20.11 (413.84) | 6.06 (4.3) | 2.6 (2.19) |
|  | angina pectoris | 78 | 3.29 (2.52 - 4.3) | 3.29 (85.43) | 2.57 (2.06) | 1.36 (0.99) |
|  | atrioventricular block second degree | 38 | 27.56 (13.73 - 55.3) | 27.55 (202.53) | 6.53 (3.65) | 2.71 (2.09) |
| Skin and subcutaneous tissue disorders | skin lesion | 135 | 3.84 (3.12 - 4.73) | 3.84 (185.29) | 2.86 (2.4) | 1.51 (1.23) |
|  | solar lentigo | 57 | 12.16 (7.95 - 18.59) | 12.15 (217.99) | 5.17 (3.62) | 2.37 (1.89) |
|  | lentigo | 55 | 79.78 (31.94 - 199.3) | 79.74 (356.38) | 7.56 (3.51) | 2.92 (2.38) |
|  | actinic keratosis | 41 | 18.58 (10.43 - 33.11) | 18.58 (191.39) | 5.93 (3.66) | 2.57 (1.98) |
| Infections and infestations | meningitis cryptococcal | 48 | 17.4 (10.33 - 29.32) | 17.4 (218.2) | 5.82 (3.76) | 2.54 (2) |
|  | jc virus infection | 45 | 4.03 (2.8 - 5.8) | 4.03 (65.84) | 2.95 (2.17) | 1.56 (1.06) |
|  | papilloma viral infection | 41 | 4.13 (2.81 - 6.06) | 4.13 (61.92) | 2.99 (2.17) | 1.58 (1.06) |
| Blood and lymphatic system disorders | lymphopenia | 735 | 4.97 (4.52 - 5.46) | 4.94 (1375.84) | 3.34 (3.09) | 1.74 (1.61) |
|  | leukopenia | 256 | 3.85 (3.31 - 4.48) | 3.84 (352.06) | 2.86 (2.52) | 1.51 (1.31) |
| Metabolism and nutrition disorders | vitamin d deficiency | 124 | 3.37 (2.72 - 4.17) | 3.37 (140.93) | 2.62 (2.19) | 1.39 (1.09) |
| Reproductive system and breast disorders | cervical dysplasia | 40 | 4.03 (2.74 - 5.93) | 4.03 (58.52) | 2.95 (2.13) | 1.56 (1.03) |

Footnotes: PT, preferred term; SOC, system organ classification. CI, confidence interval; EBGM, empirical bayesian geometric mean; EBGM05, the lower limit of 95% confidence interval of EBGM; IC, information component; IC025, the lower limit of 95% confidence interval of the IC; PRR, proportional reporting ratio; PT, preferred term; ROR, reporting odds ratio; χ^2^, Chi-squared.

**Supplementary Table 7. Safety signals for cladribine-associated adverse events in the FAERS database: primary and sensitivity analyses.**

| **PT** | **primary analysis (MS Indication)** | | **sensitivity analysis 1 (no high-risk comedications)** | | **sensitivity analysis 2 (HCP reports only)** | |
| --- | --- | --- | --- | --- | --- | --- |
|  | **Cases** | **ROR (95% CI)** | **Cases** | **ROR (95% CI)** | **Cases** | **ROR (95% CI)** |
| Lymphocyte count decreased | 336 | 6.34（5.66 - 7.10） | 328 | 6.26 (5.58 - 7.02) | 138 | 4.89 (4.11 - 5.83) |
| White blood cell count decreased | 236 | 3.82 (3.35 - 4.36) | 233 | 3.8 (3.33 - 4.34) | 55 | 2.75 (2.1 - 3.6) |
| Pneumonia | 190 | 2.85 (2.46 - 3.30) | 185 | 2.85 (2.46 - 3.31) | 37 | 1.61 (1.16 - 2.24) |
| Lymphopenia | 111 | 4.19 (3.45 - 5.08) | 108 | 4.12 (3.39 - 5.01) | 91 | 4.63 (3.74 - 5.74) |
| Lower respiratory tract infection | 71 | 6.58 (5.15 - 8.41) | 70 | 6.96 (5.43 - 8.91) | 13 | 4.24 (2.42 - 7.43) |
| Alanine aminotransferase increased | 60 | 3.61 (2.78 - 4.69) | 54 | 3.28 (2.49 - 4.31) | 51 | 4.39 (3.3 - 5.83) |
| Aspartate aminotransferase increased | 48 | 4.60 (3.43 - 6.16) | 44 | 4.24 (3.12 - 5.76) | 42 | 6.06 (4.41 - 8.32) |
| Nephrolithiasis | 48 | 2.64 (1.98 - 3.52) | 46 | 2.58 (1.92 - 3.47) | 6 | 1.2 (0.54 - 2.69) |
| Kidney infection | 39 | 4.08 (2.95 - 5.64) | 39 | 4.19 (3.03 - 5.79) | 3 | 1.22 (0.39 - 3.81) |
| Platelet count decreased | 37 | 3.84 (2.76 - 5.35) | 36 | 3.78 (2.7 - 5.29) | 18 | 3.97 (2.47 - 6.4) |
| Hypothyroidism | 32 | 5.04 (3.52 - 7.23) | 32 | 5.1 (3.56 - 7.31) | 12 | 4.85 (2.7 - 8.72) |
| Leukopenia | 32 | 2.89 (2.03 - 4.12) | 31 | 2.84 (1.98 - 4.07) | 26 | 3.37 (2.27 - 5) |
| Neutrophil count decreased | 31 | 6.13 (4.24 - 8.87) | 31 | 6.24 (4.32 - 9.03) | 18 | 6.16 (3.8 - 9.99) |
| Diverticulitis | 30 | 5.55 (3.82 - 8.06) | 29 | 5.4 (3.7 - 7.89) | 4 | 2.34 (0.86 - 6.33) |
| Drug-induced liver injury | 22 | 5.94 (3.84 - 9.19) | 18 | 4.82 (2.98 - 7.78) | 18 | 7.6 (4.67 - 12.37) |
| Haematochezia | 22 | 5.09 (3.30 - 7.86) | 22 | 5.2 (3.37 - 8.03) | 0 | - |
| Rheumatoid arthritis | 21 | 5.64 (3.61 - 8.82) | 21 | 5.69 (3.64 - 8.9) | 6 | 7.58 (3.26 - 17.6) |
| Hypophagia | 19 | 5.80 (3.63 - 9.28) | 19 | 5.83 (3.65 - 9.33) | 3 | 4.61 (1.43 - 14.82) |
| Thrombocytopenia | 19 | 3.64 (2.30 - 5.79) | 18 | 3.46 (2.15 - 5.56) | 15 | 3.7 (2.2 - 6.23) |
| Dysmenorrhoea | 17 | 13.82 (8.19 - 23.33) | 17 | 14.3 (8.46 - 24.19) | 15 | 55.97 (28.42 - 110.22) |
| Blood potassium decreased | 17 | 3.15 (1.93 - 5.12) | 17 | 3.15 (1.94 - 5.13) | 2 | 1.24 (0.31 - 5.02) |
| Cyst | 15 | 4.20 (2.49 - 7.09) | 15 | 4.3 (2.55 - 7.25) | 4 | 5.05 (1.83 - 13.93) |
| Blood bilirubin increased | 14 | 4.03 (2.35 - 6.91) | 13 | 3.79 (2.17 - 6.63) | 13 | 5.32 (3.03 - 9.36) |
| Autoimmune thyroiditis | 12 | 4.29 (2.39 - 7.69) | 11 | 3.89 (2.12 - 7.15) | 8 | 5.01 (2.44 - 10.27) |
| Eye haemorrhage | 11 | 6.88 (3.69 - 12.8) | 11 | 7.12 (3.82 - 13.28) | 3 | 8.15 (2.47 - 26.95) |
| Unresponsive to stimuli | 11 | 4.61 (2.50 - 8.51) | 11 | 4.59 (2.49 - 8.47) | 2 | 1.91 (0.47 - 7.78) |
| Premature baby | 10 | 7.47 (3.88 - 14.38) | 10 | 7.53 (3.91 - 14.49) | 6 | 13.69 (5.71 - 32.83) |
| Angioedema | 9 | 4.24 (2.16 - 8.32) | 8 | 3.78 (1.85 - 7.71) | 6 | 4.66 (2.04 - 10.66) |
| Acute myocardial infarction | 9 | 3.70 (1.89 - 7.25) | 9 | 3.71 (1.89 - 7.27) | 5 | 3.21 (1.31 - 7.88) |
| Complication of pregnancy | 8 | 16.25 (7.49 - 35.27) | 8 | 16.18 (7.46 - 35.13) | 4 | 47.13 (13.29 - 167.08) |
| Graves' disease | 8 | 10.40 (4.93 - 21.94) | 8 | 11.77 (5.54 - 25) | 6 | 12.86 (5.38 - 30.71) |
| Myelodysplastic syndrome | 8 | 8.81 (4.21 - 18.45) | 8 | 8.78 (4.19 - 18.37) | 6 | 11.17 (4.72 - 26.43) |
| Brain oedema | 8 | 6.12 (2.96 - 12.63) | 8 | 6.09 (2.95 - 12.58) | 3 | 5.44 (1.68 - 17.6) |
| Cervical dysplasia | 8 | 5.00 (2.43 - 10.26) | 8 | 4.98 (2.43 - 10.22) | 5 | 3.84 (1.56 - 9.46) |
| Uveitis | 8 | 4.86 (2.37 - 9.97) | 8 | 4.93 (2.4 - 10.12) | 2 | 2.28 (0.56 - 9.32) |
| Faeces discoloured | 8 | 4.77 (2.33 - 9.78) | 8 | 4.84 (2.36 - 9.93) | 2 | 5.05 (1.2 - 21.19) |
| Pneumonia viral | 7 | 12.64 (5.62 - 28.40) | 7 | 13.33 (5.91 - 30.07) | 1 | 5.05 (0.66 - 38.38) |
| Completed suicide | 7 | 7.46 (3.41 - 16.31) | 7 | 7.43 (3.4 - 16.24) | 5 | 8.03 (3.18 - 20.27) |
| Subcutaneous abscess | 7 | 6.23 (2.87 - 13.54) | 7 | 6.47 (2.98 - 14.08) | 3 | 6.42 (1.97 - 20.96) |
| Pneumonia bacterial | 7 | 4.59 (2.13 - 9.89) | 7 | 4.72 (2.19 - 10.17) | 2 | 3.21 (0.78 - 13.25) |
| Retinal detachment | 7 | 4.37 (2.03 - 9.40) | 7 | 4.44 (2.06 - 9.56) | 2 | 5.65 (1.34 - 23.87) |
| Pertussis | 6 | 22.94 (9.04 - 58.18) | 5 | 23.11 (8.32 - 64.18) | 1 | 8.83 (1.1 - 70.62) |
| Polyarthritis | 6 | 16.25 (6.64 - 39.75) | 6 | 16.18 (6.61 - 39.59) | 2 | 9.42 (2.15 - 41.21) |
| Metastases to central nervous system | 6 | 9.28 (3.95 - 21.84) | 6 | 9.25 (3.93 - 21.75) | 6 | 14.63 (6.07 - 35.26) |
| Blood creatine phosphokinase increased | 6 | 6.96 (3.00 - 16.16) | 5 | 5.88 (2.35 - 14.7) | 4 | 7.25 (2.59 - 20.3) |
| Hepatitis c | 6 | 6.39 (2.76 - 14.79) | 6 | 6.37 (2.75 - 14.73) | 1 | 17.66 (1.97 - 158.05) |
| Synovial cyst | 6 | 5.34 (2.32 - 12.28) | 6 | 5.71 (2.48 - 13.16) | 1 | 2.62 (0.36 - 19.26) |
| Arthritis infective | 6 | 4.94 (2.15 - 11.32) | 6 | 5.04 (2.2 - 11.58) | 1 | 3.36 (0.45 - 25.01) |
| Pericarditis | 6 | 4.75 (2.08 - 10.89) | 6 | 4.74 (2.07 - 10.85) | 4 | 6.15 (2.21 - 17.08) |
| Plasma cell myeloma | 6 | 4.59 (2.00 - 10.5) | 6 | 4.68 (2.04 - 10.72) | 3 | - 1. 1.68 - 17.6) |

***Abbreviations:* PT, Preferred Term; CI, confidence interval; HCP, healthcare professional; MS, multiple sclerosis; ROR, reporting odds ratio.
*Notes:***

- **The primary analysis was restricted to reports with Multiple Sclerosis as the indicated condition. Sensitivity Analysis 1 excluded cases with concomitant use of frequent and high-risk medications. Sensitivity Analysis 2 was restricted to reports submitted exclusively by healthcare professionals.**
- **A positive signal was defined as meeting both of the following criteria: a case count ≥ 3 and the lower limit of the 95% confidence interval (CI) for the Reporting Odds Ratio (ROR) > 1.**
- **Red text: PT does not meet the positive signal criteria (case count ≥ 3 and lower limit of the 95% CI > 1).**
