# Supplementary material for: Post-marketing safety profile of cladribine in multiple sclerosis: a disproportionality analysis based on the FDA adverse event reporting system
Source: Int J Clin Pharm. 2025 Nov 15;48(2):623–35. doi: 10.1007/s11096-025-02041-8 (PMC12992368; doi:10.1007/s11096-025-02041-8)
Supplement: Supplementary file 2 — Supplementary file2 (DOCX 20 KB) [file 11096_2025_2041_MOESM2_ESM.docx]

# Disproportionality Analysis Code

## Purpose

This code performs disproportionality analysis for drug safety signal detection using four standard methods.

## Required Input Data Format

The input Excel file should contain these columns for each drug-event pair:

- a: Reports for target drug and target adverse event

- b: Reports for target drug and other adverse events

- c: Reports for other drugs and target adverse event

- d: Reports for other drugs and other adverse events

## Methods Implemented

1. Reporting Odds Ratio (ROR) with 95% CI

2. Proportional Reporting Ratio (PRR) with 95% CI and chi-square

3. Empirical Bayes Geometric Mean (EBGM) - MGPS method

4. Bayesian Confidence Propagation Neural Network (BCPNN)

## Signal Criteria

- ROR signal: lower 95% CI > 1 and ≥3 reports

- PRR signal: PRR ≥ 2, chi-square ≥ 4, and ≥3 reports

- EBGM signal: EBGM05 > 2

- BCPNN signal: IC025 > 0

# Methods: ROR, PRR, EBGM (MGPS), BCPNN

library(dplyr)

library(data.table)

library(readxl)

library(openxlsx)

# ==================== DATA INPUT ====================

# Read the dataset for analysis

# File contains columns: a, b, c, d for 2x2 contingency table

# a: reports for target drug and target adverse event

# b: reports for target drug and other adverse events

# c: reports for other drugs and target adverse event

# d: reports for other drugs and other adverse events

adr_data <- read_xlsx("data/ADR_Analysis_Data.xlsx") %>% data.table()

# ==================== CALCULATIONS ====================

if (nrow(adr_data) > 0) {

# 1. Reporting Odds Ratio (ROR) with 95% CI

adr_data[, ROR := (a * d) / (b * c)]

adr_data[, ROR_lower := exp(log(ROR) - 1.96 * sqrt(1/a + 1/b + 1/c + 1/d))]

adr_data[, ROR_upper := exp(log(ROR) + 1.96 * sqrt(1/a + 1/b + 1/c + 1/d))]

# 2. Proportional Reporting Ratio (PRR) with 95% CI and Chi-square

adr_data[, PRR := (a / (a + b)) / (c / (c + d))]

adr_data[, PRR_lower := exp(log(PRR) - 1.96 * sqrt(1/a - 1/(a + b) + 1/c - 1/(c + d)))]

adr_data[, PRR_upper := exp(log(PRR) + 1.96 * sqrt(1/a - 1/(a + b) + 1/c - 1/(c + d)))]

adr_data[, chi_square := ((a * d - b * c)^2 * (a + b + c + d)) / ((a + b) * (c + d) * (a + c) * (b + d))]

# 3. Empirical Bayes Geometric Mean (EBGM) for MGPS

adr_data[, EBGM := (a * (a + b + c + d)) / ((a + b) * (a + c))]

adr_data[, EBGM_05 := exp(log(EBGM) - 1.64 * sqrt(1/a + 1/b + 1/c + 1/d))]

# 4. Bayesian Confidence Propagation Neural Network (BCPNN)

adr_data[, IC := log2((a * (a + b + c + d)) / ((a + c) * (a + b)))]

adr_data[, gamma_prior := ((a + b + c + d + 2)^2) / ((a + b + 1) * (a + c + 1))]

adr_data[, E_IC := log2(((a + 1) * (a + b + c + d + 2)^2) /

((a + b + c + d + gamma_prior) * (a + b + 1) * (a + c + 1)))]

adr_data[, V_IC := (1 / log(2)^2) *

((a + b + c + d - 3 + gamma_prior) / (3 * (1 + a + b + c + d + gamma_prior)) +

(b + c + d + 1) / ((a + b + 1) * (a + b + c + d + 2)) +

(b + c + d + 1) / ((a + c + 1) * (a + b + c + d + 3)))]

adr_data[, IC_SD := sqrt(V_IC)]

adr_data[, IC_025 := IC - 2 * IC_SD]

}

# ==================== RESULTS FORMATTING ====================

# Select and arrange columns

results <- data.frame(adr_data) %>%

dplyr::select(

PT_term, a, b, c, d,

ROR, ROR_lower, ROR_upper,

PRR, chi_square, PRR_lower, PRR_upper,

EBGM, EBGM_05,

IC, IC_025,

pt_name, soc_name

) %>%

arrange(desc(a))

# Round numerical values

numeric_columns <- c("ROR", "ROR_lower", "ROR_upper", "PRR", "PRR_lower",

"PRR_upper", "chi_square", "EBGM", "EBGM_05", "IC", "IC_025")

results[numeric_columns] <- lapply(results[numeric_columns], function(x) round(x, 2))

# Create combined display columns

results <- results %>%

mutate(

ROR_95CI = paste0(ROR, " (", ROR_lower, "-", ROR_upper, ")"),

PRR_chi = paste0(PRR, " (", chi_square, ")"),

EBGM_result = paste0(EBGM, " (", EBGM_05, ")"),

IC_result = paste0(IC, " (", IC_025, ")")

)

# ==================== SIGNAL DETECTION ====================

results <- results %>%

mutate(

ROR_signal = case_when(

ROR_lower > 1 & a >= 3 ~ "Yes",

TRUE ~ "No"),

PRR_signal = case_when(

PRR >= 2 & a >= 3 & chi_square >= 4 ~ "Yes",

TRUE ~ "No"),

EBGM_signal = case_when(

EBGM_05 > 2 ~ "Yes",

TRUE ~ "No"),

BCPNN_signal = case_when(

IC_025 > 0 ~ "Yes",

TRUE ~ "No")

)

# ==================== OUTPUT ====================

# Save results

write.xlsx(results, file = "results/Disproportionality_Analysis_Results.xlsx")

cat("Disproportionality analysis completed successfully!\n")

cat("Four methods implemented: ROR, PRR, EBGM (MGPS), BCPNN\n")

cat("Results saved to: results/Disproportionality_Analysis_Results.xlsx\n")

#CHI-SQUARE AND FISHER'S EXACT TESTS

# Statistical comparison of adverse event frequencies between sex groups

# Used for identifying significant differences in AE reporting patterns

library(readxl)

library(writexl)

# ==================== DATA INPUT ====================

# Read the dataset containing adverse event counts by sex

# Expected columns:

# - Group1: Number of reports for each AE in Group1s

# - Group2: Number of reports for each AE in Group2s

input_data <- read_excel("data/Adverse_Events_by_Sex.xlsx")

# ==================== STUDY POPULATION ====================

# Total number of reports in each sex group

total_Group1s <- 1000 # Total Group1 population in the study

total_Group2s <- 10000 # Total Group2 population in the study

# ==================== STATISTICAL ANALYSIS FUNCTION ====================

calculate_sex_comparison_pvalue <- function(Group1_ae_count, Group2_ae_count) {

"""

Calculate p-value for sex-based comparison of adverse event frequencies

Automatically selects appropriate test based on expected frequencies

Parameters:

Group1_ae_count: Number of AE reports in Group1s

Group2_ae_count: Number of AE reports in Group2s

Returns:

List containing formatted p-value and test method used

"""

# Calculate complementary counts

Group1_other <- total_Group1s - Group1_ae_count

Group2_other <- total_Group2s - Group2_ae_count

# Create 2x2 contingency table

contingency_table <- matrix(c(Group1_ae_count, Group1_other,

Group2_ae_count, Group2_other),

nrow = 2,

dimnames = list(Sex = c("Group1", "Group2"),

Outcome = c("AE", "No_AE")))

# Check if any cell count is zero

if (any(contingency_table == 0)) {

return(list(p_value = NA,

test_method = "Not applicable - zero count"))

}

# Calculate expected frequencies

total_obs <- sum(contingency_table)

expected_freq <- outer(rowSums(contingency_table),

colSums(contingency_table)) / total_obs

# Select test based on expected frequencies

# Use Fisher's exact test if any expected frequency < 5

if (any(expected_freq < 5)) {

test_result <- fisher.test(contingency_table)

return(list(p_value = test_result$p.value,

test_method = "Fisher's exact test"))

} else {

# Use Chi-square test for larger expected frequencies

test_result <- chisq.test(contingency_table)

return(list(p_value = test_result$p.value,

test_method = "Chi-square test"))

}}

# ==================== APPLY ANALYSIS ====================

# Initialize columns for results

input_data$P_value <- NA

input_data$Test_method <- NA

input_data$Formatted_p_value <- NA

# Apply statistical test to each adverse event

for (i in 1:nrow(input_data)) {

Group1_count <- input_data$Group1[i]

Group2_count <- input_data$Group2[i]

result <- calculate_sex_comparison_pvalue(Group1_count, Group2_count)

input_data$P_value[i] <- result$p_value

input_data$Test_method[i] <- result$test_method

# Format p-value for reporting

if (is.na(result$p_value)) {

input_data$Formatted_p_value[i] <- "Not applicable"

} else if (result$p_value < 0.001) {

input_data$Formatted_p_value[i] <- "P < 0.001"

} else if (result$p_value < 0.01) {

input_data$Formatted_p_value[i] <- "P < 0.01"

} else if (result$p_value < 0.05) {

input_data$Formatted_p_value[i] <- "P < 0.05"

} else {

input_data$Formatted_p_value[i] <- sprintf("P = %.3f", result$p_value)}}

# ==================== RESULTS SUMMARY ====================

# Count tests used

test_summary <- table(input_data$Test_method, useNA = "ifany")

cat("Statistical tests used:\n")

print(test_summary)

# Count significant findings (P < 0.05)

significant_count <- sum(input_data$P_value < 0.05, na.rm = TRUE)

total_tests <- sum(!is.na(input_data$P_value))

cat(sprintf("\nSignificant findings (P < 0.05): %d out of %d tests (%.1f%%)\n",

significant_count, total_tests,

(significant_count/total_tests)*100))

# ==================== OUTPUT ====================

# Save complete results

write_xlsx(input_data, "results/Sex_Comparison_Statistical_Analysis.xlsx")

# Create simplified version for reporting (optional)

reporting_data <- input_data[, c("Adverse_Event", "Group1", "Group2",

"Formatted_p_value", "Test_method")]

write_xlsx(reporting_data, "results/Sex_Comparison_Summary_Table.xlsx")

cat("\nAnalysis completed successfully!\n")

cat("Results saved to:\n")

cat("- results/Sex_Comparison_Statistical_Analysis.xlsx (complete results)\n")

cat("- results/Sex_Comparison_Summary_Table.xlsx (reporting table)\n")
